# Diagnostic reliability and accuracy of the hydraulic contrast lift protocol in the radiographic detection of sinus lift and perforation: ex vivo randomized split-mouth study in an ovine model

**DOI:** 10.1038/s41405-024-00188-6

**Published:** 2024-01-31

**Authors:** Mohamed A. Youssef, Nadine von Krockow, Jacqueline A. Pfaff

**Affiliations:** 1 Master of Oral Implantology program, J. W. Goethe University, Theodor-Stern-Kai 7, Haus 29, 60596, Frankfurt am Main, Hessen Germany; 2 Private practice focused on implant supported rehabilitation, Montreal, Quebec Canada; 3 Department of Postgraduate Education, J. W. Goethe University, Theodor-Stern-Kai 7, Haus 29, 60596 Frankfurt am Main, Hessen Germany; 4 Oral Surgeon in Private Practice, Frankfurt am Main, Hessen Germany; 5 Oral Surgeon, Salzburg, Austria

**Keywords:** Dental implants, Oral surgery, Periapical radiographs

## Abstract

**Objectives:**

Assessing the diagnostic reliability, validity, and accuracy of the hydraulic contrast lift protocol during transcrestal sinus floor elevation in detecting the lift and perforation of the sinus membrane before graft material application and assessing the effect of its use on the operator’s diagnostic confidence.

**Material and methods:**

A single-blind randomized split-mouth study on fresh refrigerated sheep heads. The first intervention consisted of injecting 0.5 ml iodinated contrast medium on the test side and 0.5 ml saline on the control side. In the second intervention artificial sinus membrane perforations were created followed by injecting 0.5 ml iodinated contrast medium on the test side and 0.5 ml saline on the control side. Intraoperative periapical radiographs were taken for both interventions. The resulting 40 radiographs were assessed by 10 examiners to provide interpretations and confidence ratings. The primary endpoints were diagnostic reliability, validity, accuracy, and perceived diagnostic confidence.

**Results:**

In the hydraulic contrast lift protocol, the detection rate was 99% for sinus elevations and 98% for perforations, the saline protocol yielded a detection rate of 28% and 20% respectively. The hydraulic contrast lift protocol demonstrated a high level of inter-rater agreement for the diagnosis of elevations (*p* < 0.001) and perforations (*p* < 0.001), strong diagnostic validity for the diagnosis of elevations (*p* < 0.001) and perforations (*p* < 0.001), high sensitivity and specificity (*p* < 0.001) and higher mean diagnostic confidence ratings for both interventions when compared to the saline protocol (*p* < 0.001). The difference between the predicted probability for correct diagnosis of the hydraulic contrast lift protocol and the saline protocol was significant (*p* < 0.001) for the detection of both elevations and perforations.

**Conclusion:**

Following the hydraulic contrast lift protocol, the use of a radiographic contrast medium can reliably confirm sinus membrane lift and detect perforation during transcrestal sinus floor elevation prior to bone graft application in addition to improving the diagnostic confidence of the operator while relying on periapical radiographs.

## Introduction

Loss of teeth and old age are identified as two important factors in relation to maxillary sinus pneumatization and reduced residual maxillary bone [1]. In the posterior maxilla, sinus augmentation was reported for as high as 95% of the implants placed when the residual ridge height was less than 8 mm and around half of the implants placed in that area were associated with a type of sinus floor elevation (SFE) procedure regardless of the remaining bone height [2]. The first technique described for SFE was the lateral window approach by Tatum and Boyne in 1977 and by James in 1980 using a modified Caldwell-Luc Technique [3]. When performing a maxillary sinus augmentation procedure, the most common complication is Schneiderian membrane perforation [4] and by preventing this complication, other subsequent sequalae including sinusitis, epistaxis, oroantral communication, nasal cavity penetration and maxillary ostium obstruction can be reduced or avoided. Several studies have reported a lower frequency of Schneiderian membrane perforation when performing a transcrestal sinus floor elevation (TSFE) as opposed to the traditional lateral sinus floor elevation (LSFE) [5–9]. Among the merits of LSFE are adequate accessibility, visibility, and ability to achieve good augmentation height. On the other hand, the TSFE technique offers several benefits, including a more conservative and less invasive approach. It eliminates the need for an additional surgical access (lateral window) when grafting and implant placement are performed simultaneously. Furthermore, TSFE is associated with fewer post-operative complications, enhanced patient comfort, and improved containment and blood supply to the grafted site by preserving the lateral wall [10, 11]. Sotirakis and Gonshor [10] introduced the hydraulic sinus floor elevation (HSFE) as a modification of the TSFE technique. This modified approach aimed to combine the benefits of TSFE, including the advantages mentioned earlier, with a comparable increase in augmentation height as achieved through LSFE. Consequently, the only remaining significant drawback of the TFSE was its limited visibility, which led to its characterization as a “blind technique” [12, 13].

### Purpose

The lack of an objective sign of Schneiderian membrane integrity before the graft material is applied leads to a higher potential for undetected minor or major membrane perforations representing a risk that cannot be completely eliminated. This uncertainty could be a reason why some clinicians still prefer the more invasive LSFE. This problem creates a need to enhance the detection of membrane tears when performing such procedures before, as opposed to after, the graft material application. For the operator, eliminating the uncertainty could encourage the use of a more conservative technique improving patients’ comfort without compromising their safety. The purpose of this study is to resolve this uncertainty and provide the clinicians, through a non-invasive method, with clear criteria for reliable detection of perforations during TSFE.

### Aim

The aim of this study is to validate a modified HSFE technique namely the hydraulic contrast lift (HCL) protocol, that incorporates an iodinated contrast medium instead of saline, by verifying the effectiveness of this technique in confirming sinus membrane lift and identifying membrane perforations, rendering it a safer and more predictable procedure by avoiding the expression of foreign body material into the lumen of the maxillary sinus.

### Objectives of the study

Assessing the diagnostic reliability, validity, and accuracy of the HCL protocol as a diagnostic tool during TSFE to confirm the lift and detect the perforation of the sinus membrane before graft material application.

Assessing the effect of using this protocol on the diagnostic confidence of the operator.

### Study question

Following the HCL protocol, can the use of a radiographic contrast medium during TSFE reliably and accurately identify and differentiate between a successfully lifted and a perforated sinus membrane intra-operatively while relying on periapical radiographs?

### Hypothesis

We hyposthesize that using the HCL protocol, where an iodinated contrast medium is used for sinus membrane lifting, we can reliably and accurately confirm sinus membrane lift or detect membrane perforation intra-operatively before graft material application in comparison to the current technique using saline.

### Background and review of the literature

The first publication to describe a Transcrestal approach for sinus floor elevation was by Tatum in 1986 where osteotomes were used in direct contact with the sinus membrane to lift it [14] followed by Summers in 1994 where osteotomes were malleted with the aim to penetrate the sinus floor while displacing a layer of bone within the sinus lumen without perforating the Schneiderian membrane [15]. Several techniques were later described as modifications of the osteotome original approach such as using a balloon [16, 17] or using hydraulic pressure to lift the Schneiderian membrane [10, 18]. The recommendation for the traditional osteotome technique is to aim only for a bone gain of 3–4 mm bone in the vertical height [19, 20] which limits the indications and clinical use of this technique. HSFE has been first described in 2005 as a variant of TSFE that can reduce the incidence of perforation of the Schneiderian membrane associated with TSFE especially in cases where anatomical restrictions are present [10, 18]. HSFE was later suggested as a technique modification used to perform SFE through a lateral approach [21]. The rational for using HSFE was to apply uniform pressure on the Schneiderian membrane during the lift, hence reducing the possibility for its perforation [22].

### Complications following TSFE

Two systematic reviews in which most of the studies included used the osteotome technique reported that the most common surgical complication was the Schneiderian membrane perforation with an incidence ranging between 0% to 21% and 0% to 26% [23, 24], which in turn can lead to the development of sinusitis, epistaxis, exfoliation of graft particles from the nose, or a patent oral-antral communication. The most reported postoperative complications other than membrane perforation include nosebleed and infection followed by paroxysmal vertigo, haemorrhage, hematoma, cover screw loosening and implant displacement into the sinus [20, 23–25]. TSFE could lead to rare complications, more specifically a case report described the occurrence of conjunctival chemosis [26] and another described a brain abscess as a result of the procedure [27]. Intrusion of graft material into the sinus lumen is associated with the formation of fungus ball (aspergilloma) within the maxillary sinus that require additional surgical intervention for removal [28, 29].

### Limitations in detection of schneiderian membrane perforations

All the variants of TFSE are performed, in most part, blindly through a minimal access hole [12, 13]. When performing TSFE, perforations can go undetected, this was highlighted in a human cadaver study where the osteotome technique was used and perforations were detected in 6 out of 25 sites (24%) after having removed the lateral nasal wall to inspect the membrane directly and reveal the perforations [30]. To verify membrane integrity or detect perforation, the Valsalva manoeuvre has traditionally been identified as the most commonly used test while performing a TSFE procedure [8, 9, 31, 32], unfortunately, it is widely considered to be a subjective test. Other studies described the combination of the Valsalva manoeuvre along with tactile feedback to feel the elasticity of the Schneiderian membrane [33], the nose-blow test [34], and the mirror fog up test, where the patient inhales and exhales while a mirror is placed under the osteotomy, if fog is detected it is considered a sign for presence of membrane perforation [35]. Detection of membrane perforation relying only on such tests intraoperatively can be unreliable and the rate of detection can vary from one clinician to another. The shortcomings of the Valsalva manoeuvre as an intraoperative test can be summarized as follows: low specificity and limited sensitivity, inaccurate detection, patient discomfort due to forceful exhalation against a closed airway, and operator dependency where less experienced clinicians may have difficulties in correctly interpreting the results. To overcome the lack of visibility during TSFE, attempts were made to develop innovative methods to identify membrane perforations other than the Valsalva manoeuvre and its variants, including, the direct visualization of the sinus membrane during TSFE using endoscopy [36–38], monitoring of pressure change during TSFE procedure using the Jeder-System [39], the use of operating microscope or micro-camera [40], and the use of a contrast medium [22, 41].

### Contrast medium

**Fig. 1:**
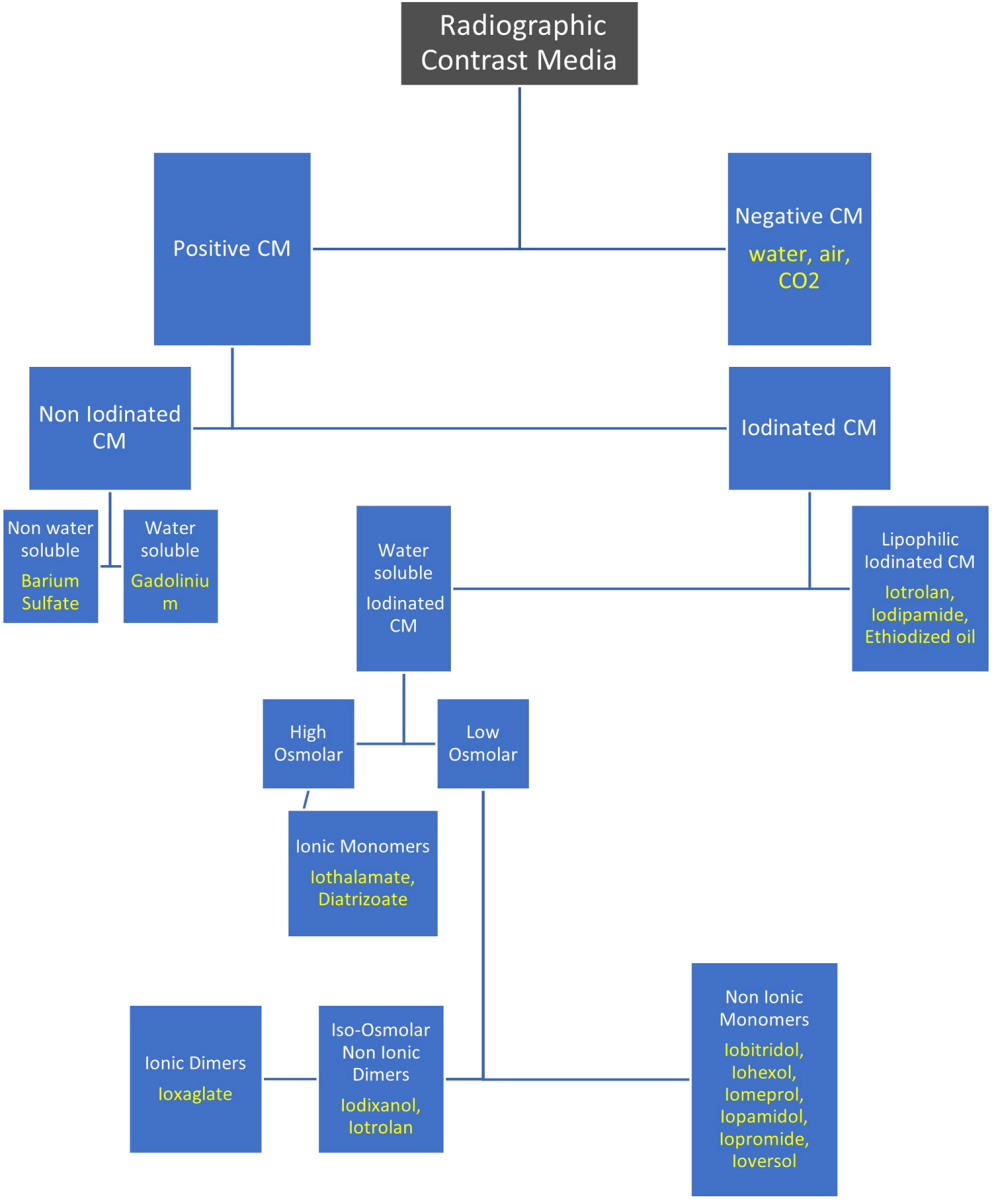
Classification of contrast media. Classification of radiographic contrast media, examples for each class are written in yellow.

Radiographic contrast media were used historically in the radiographic study of the maxillary sinuses where it was injected into the lumen of the sinus to fill it completely and the drainage of the contrast medium occurred over the period of a few days [42]. Contrast media are classified as positive and negative contrast agents. Positive contrast agents block the passage of x-rays through them more than the soft tissues in the body and hence appear more radiopaque. Orally administered positive contrast agents are used for studies of the gastrointestinal tract, they are divided into water soluble and non water soluble groups. The water soluble group is iodine based and the non water soluble group is made from a suspension of Barium Sulphate [43, 44]. Iodine-based contrast media are divided into 4 groups: high-osmolar ionic monomers, low-osmolar ionic dimers, low-osmolar non-ionic monomers, and iso-osmolar non-ionic dimers [45] (Fig. 1). Non ionic low-osmolar contrast media have lower adverse effects and toxicity compared to high-osmolar ionic agents [46–48].

Aspiration of contrast media administered via the oral route is a common complication which occurs in up to 22% of fluoroscopic examinations [48]. Contrast agent aspiration can cause serious complications such as chemical pneumonitis and acute respiratory distress syndrome these reactions are more prominent with the use of high-osmolar contrast media where the high osmolality drives water into the alveoli, leading to pulmonary oedema [44]. Barium sulphate aspiration can be quite dangerous with a risk of death close to 40% [49]. Iohexol is a non ionic low-osmolar agent that has an osmolality similar to that of the plasma, this low osmolality in addition to its inert nature and water solubility reduce the risk of contrast-induced pneumonitis in case of aspiration [50]. The palatability of iohexol is an added benefit making it one of the preferred orally administered contrast agents [44, 51].

### Animal model

**Fig. 2:**
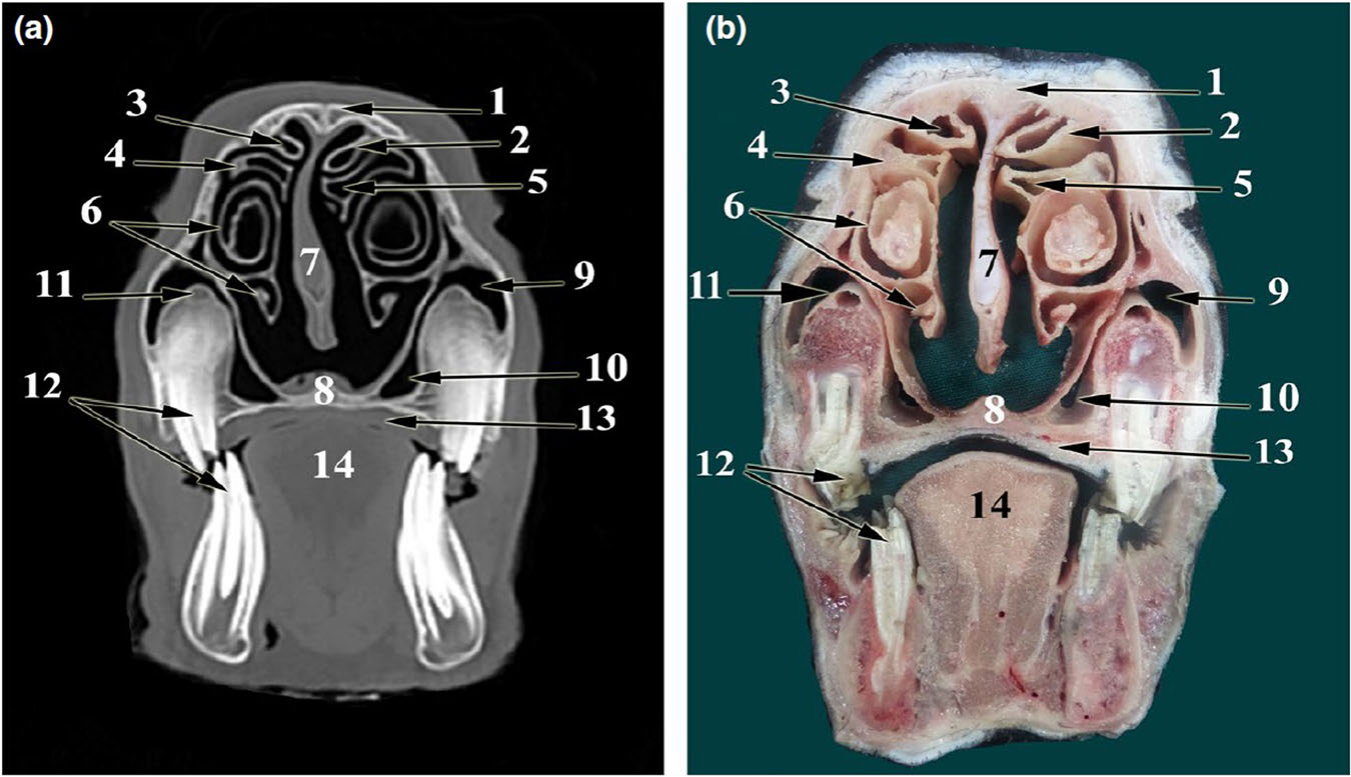
CT and cross-sectional images of sheep skull. CT scan images (**a**) and cross‐sectional images (**b**) at the level of the 2nd molar tooth 1 cm rostral to the medial canthus (**a**, **b**). Nasal bone (1), dorsal nasal concha (2), dorsal conchal sinus (3), middle nasal concha (4), middle conchal sinus (5), dorsal and ventral spiral lamellae of the ventral nasal concha (6), nasal septum (7), palatine process of maxilla (8), lateral chamber of the maxillary sinus (9), medial chamber of the maxillary sinus (10), infraorbital canal (11), dorsal and ventral 2nd molar teeth (12), hard palate (13), tongue (14). Image reproduced with permission from Awaad et al, ‘Surgical anatomy of the nasal and paranasal sinuses in Egyptian native sheep (Ovis aries) using computed tomography and cross sectioning’, Anat Histol Embryo, 2019, Wiley [54].

**Fig. 3:**
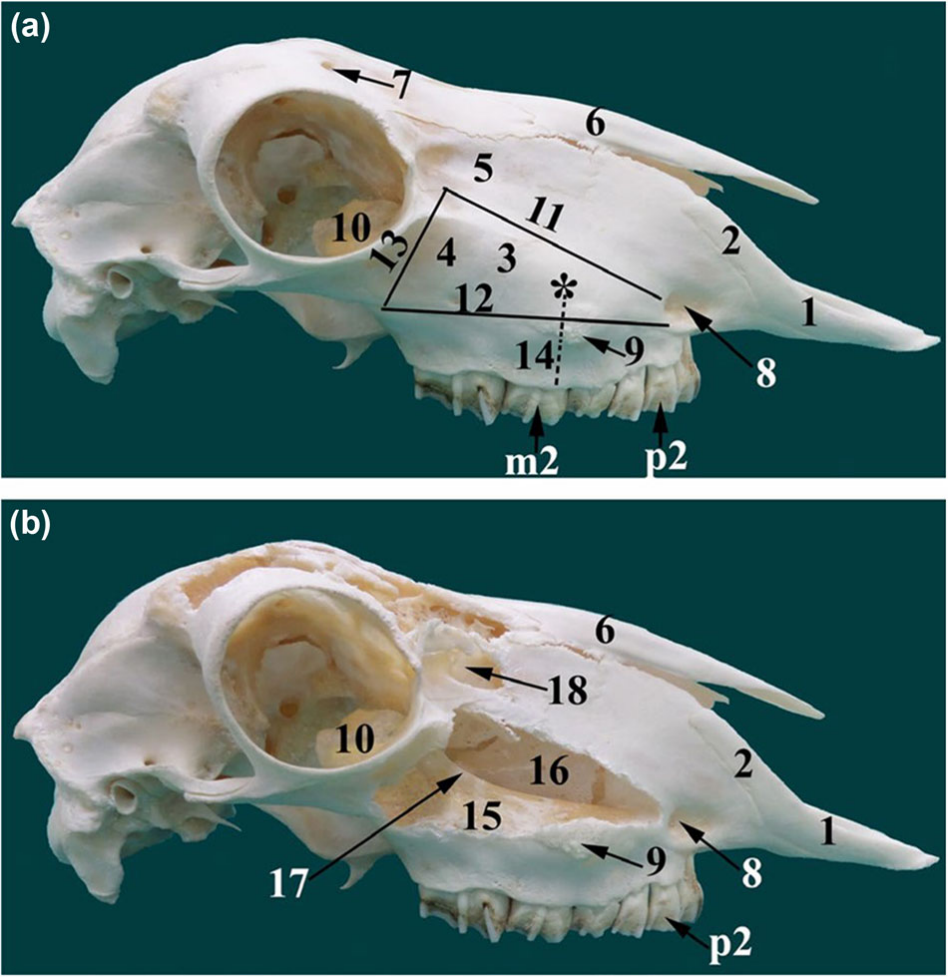
Lateral view of sheep skull. Lateral view of the sheep skull (**a**, **b**) showing the maxillary sinuses. Incisive bone (1), nasal process (2) of (1), maxilla (3), zygomatic bone (4), lacrimal bone (5), nasal bone (6), supraorbital foramen (7), infraorbital foramen (8), facial tubercle (9), lacrimal bulla (10), dorsal limit of the maxillary sinus (11), ventral limit of the maxillary sinus (12), caudal limit of the maxillary sinus (13), the trephination site of the maxillary sinus (14*), lateral chamber of the maxillary sinus (15), medial chamber of the maxillary sinus (16), infraorbital canal (17), lacrimal sinus (18), second pre‐molar tooth (p2), second molar tooth (m2). Image reproduced with permission from Awaad et al. ‘Surgical anatomy of the nasal and paranasal sinuses in Egyptian native sheep (Ovis aries) using computed tomography and cross sectioning’, Anat Histol Embryo, 2019, Wiley [54].

Since sheep had been previously identified as one of the best animal models for SFE training [52], for this study we decided to use an Ex-vivo sheep model, but first some anatomical aspects must be highlighted. In the maxillary sinus of the sheep a perpendicular structure constituted of dental roots, the bone surrounding them and the infraorbital canal at the apex of that structure incompletely divide that cavity into lateral and medial compartments [53, 54]. In a cross-sectional view of the sheep skull the medial compartment is the one that simulates the most the anatomy of a pneumatized human maxillary sinus with a triangular shape that has a wide base towards the oral cavity and a narrow layer of bone separating both cavities (Figs. 2 and 3). The lowest point among both compartments belongs in the medial chamber and was identified to be opposite to the second ovine molar [54] leading to easier surgical access from within the oral cavity to the sinus membrane (Figs. 2 and 3).

## Material and methods

### Study design

This study was performed as a single-blind randomized split-mouth study in an ex-vivo ovine model.

### Subjects, inclusion and exclusion criteria

Fresh refrigerated sheep heads sourced from a local slaughterhouse were acquired to be used in this study. These animals were not killed specifically for the research in question and were already slaughtered for the purpose of human consumption.

Sheep heads with early sinus membrane perforations, while preparing the osteotomies to access the sinus membrane, were excluded. In total, out of 15 sheep heads, 5 were excluded due to early perforations and 10 were included in the study. The number of subjects in the test and control groups were identical due to the split-mouth design.

### Intervention procedure

**Fig. 4:**
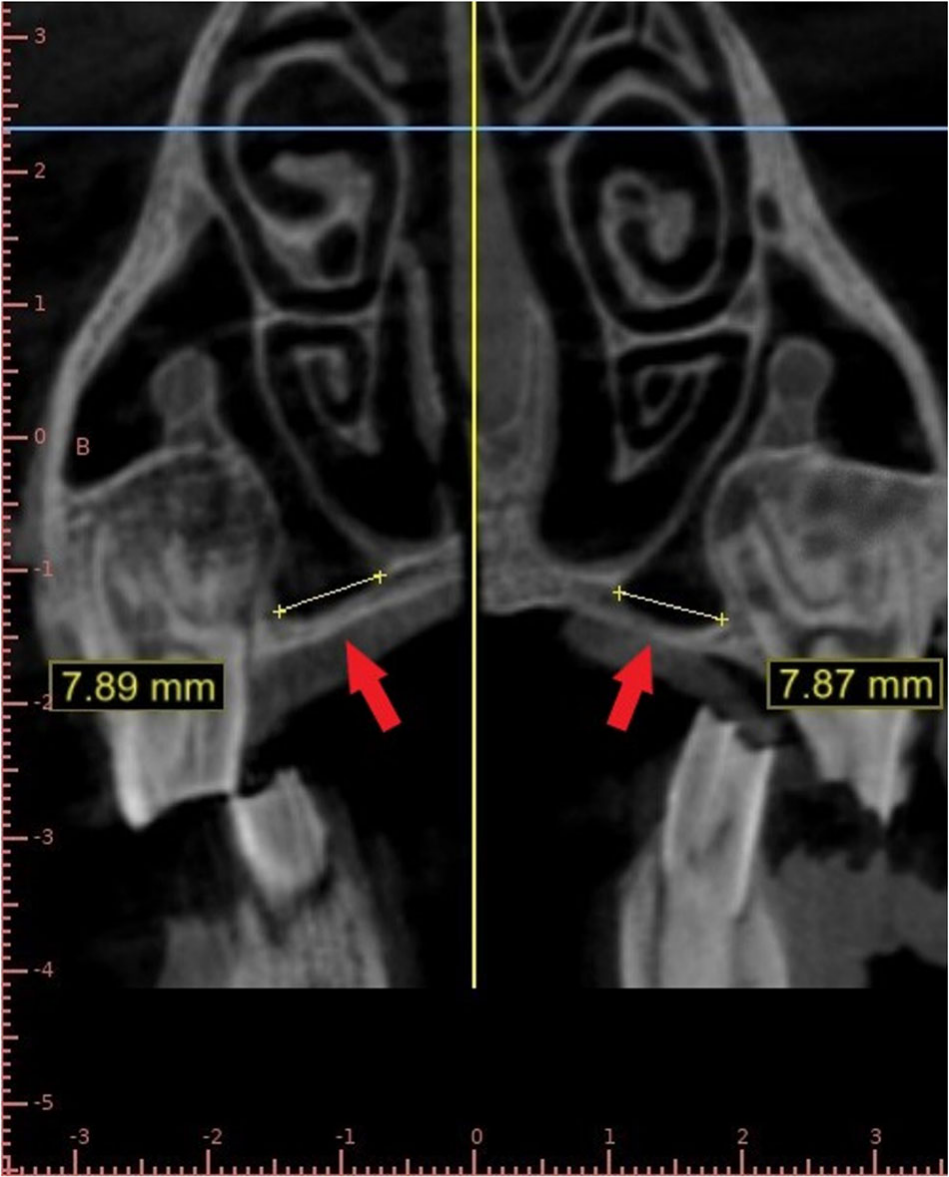
Entry point on CBCT image. Entry point shown on CBCT scan marked by the red arrows.

We used the newly developed HCL protocol [55] which consists of using an undiluted contrast agent as the lifting medium instead of saline in combination with a specifically designed apparatus that achieves multiple objectives including being self-retained in the osteotomy, allowing uninterrupted flow of the medium, providing tactile feedback to the operator indicating the volume of the injected fluid, allowing for simultaneous multiple site sinus membrane elevation in the scenario of a long span edentulous area in the posterior maxilla and preventing the leakage of the medium during its injection as well as after the injection while the diagnostic radiograph is being taken. The maxillary hard palate area 5-7 mm medial (palatal) to the first ovine molar was identified as the area most suitable to simulate an edentulous maxillary ridge in humans with sinus pneumatization. This point of entry allows access to the lowest and most accessible part of the floor of the ovine maxillary sinus (Fig. 4).

Preoperative periapical radiographs were obtained for both sides of the posterior maxilla using x-ray positioning device and a bisecting angle technique. The test side and control side were randomly assigned for each sheep head. Randomization was carried out by flipping a digital coin on the website https://www.random.org/coins/ to assign the test side, heads meant the test side would be on the right and tails meant the test side would be on the left. A full thickness mucoperiosteal flap was reflected to expose the designated points of entry, the first phase of the intervention consisted of preparing osteotomies using a large round diamond bur for the first 1–1.5 mm of drilling followed by the CAS kit drills with stoppers (Hiossen Implant Canada Inc., Markham, Ontario, Canada) to gain access to the sinus membrane while minimizing the occurrence of membrane perforations. An apparatus was specifically designed to deliver the contrast medium composed of a silicone retentive end that provides retention for a metallic injection stent within the osteotomy and seals it from the oral environment. A corresponding silicon plug was used once the contrast medium was delivered to avoid any spilling of the contrast medium while the radiographs are taken. The first set of interventions consisted of using the specifically designed apparatus along with a silicone tube and a graduated syringe to inject 0.5 ml saline solution on one side (control) and 0.5 ml Omnipaque 240 composed of 518 mg of iohexol/ml (GE Healthcare Canada Inc., Mississauga, Ontario, Canada) on the other side (test), through the established osteotomies for the sinus membrane elevation (SME) intervention. Once the respective fluid was injected, the silicone plugs were applied. Then, the first set of intra-operative periapical radiographs was taken, the plugs were removed, and the fluid was suctioned out from both sides. The second set of interventions consisted of creating an artificial perforation on each side by intentionally using a #15c scalpel blade tip to puncture the sinus membrane and create an artificial perforation (less than 3 mm) for the sinus membrane perforation (SMP) intervention. Then, a further 0.5 ml of each solution was applied to their respective sides, the plugs were applied and a second set of intraoperative periapical radiographs was taken. In this manner, the true diagnosis for each intervention was known to the main investigator prior to taking each of the radiographs. All the radiographs were taken using the same x-ray machine Belray II 097 (Takara Belmont Corporation). An overview of the study protocol can be viewed in Table 1.

**Table 1:** Study protocol with a split mouth design.

Pre-operative periapical x-rays	
Full thickness mucoperiosteal flap	
Randomization	
Osteotomies	
SME Test (T1)	SME Control (C1)
HSFE with 0.5 ml Omnipaque 240 (GE Healthcare Canada)	HSFE with 0.5 ml saline solution
*First set of intraoperative periapical x-rays*	
Removal of liquids by suction	
Artificial puncture/ perforation with #15c scalpel blade	
SMP Test (T2)	SMP Control (C2)
Injection with 0.5 ml Omnipaque 240 (GE Healthcare Canada)	Injection with 0.5 ml saline solution
*Second set of intraoperative periapical x-rays*	
Assessment of all 40 intraoperative periapical x-rays by 10 calibrated examiners	
Statistical analysis	

*SME* Sinus membrane elevation, *SMP* Sinus membrane perforation, HSFE Hydraulic sinus floor elevation.

### Type of assessment and outcome measures

Radiographic assessment of the obtained periapical radiographs was performed by 10 experienced examiners. The resulting 40 intraoperative radiographs from the first and second interventions were placed in random order using the list randomizer on the website https://www.random.org/, 10 examiners, who are dentists with more than 5 years’ experience, were then invited to assess those radiographs. Only the main investigator was aware of the group allocation and the true diagnosis for each group but not the examiners. The main investigator did not participate in the assessment of the radiographs. Before the assessment was conducted, each examiner attended a calibration session with the main investigator to explain the purpose of the study. Then, a set of normal pre-operative periapical x-rays and a coronal section of a cone beam computed tomography (CBCT) scan of the ovine maxilla were assessed to be familiarized with normal ovine radiographic anatomy. The examiners were then shown examples of periapical x-rays of the following situations: successful sinus membrane lift when using the contrast medium, successful sinus membrane lift when using saline, sinus membrane perforation when using the contrast medium and sinus membrane perforation when using saline, those x-rays were not part of the study. The examiners were blinded to each other’s assessments. The examiners were made aware that no preoperative radiographs were included, and that only those with successful sinus lift or perforations were being assessed. Then, they were asked to choose which of the following best describes their radiographic interpretation/diagnosis and to indicate their level of confidence in their chosen diagnosis with any numerical value between 1 and 5 for each of the radiographs:

1. Successful lift of the sinus membrane as evidenced by an enclosed entity/radio opacity or a well-defined entity/radio opacity that exhibits a dome or semilunar shape or ballooning/tenting of the sinus membrane.
2. Perforation of the sinus membrane as evidenced by an ill-defined entity/radio opacity or a diffuse entity/radio opacity that does not exhibit a uniform shape or has an irregular shape or that loosely follows the shape of the sinus floor and/or walls.
3. Absence of such entities or no appreciable difference from normal radiographic anatomy of the Ovine Maxilla.

An example of the questionnaire for examiners can be viewed in Appendix 2. The outcome measures of this study were successful diagnosis of sinus membrane lift and sinus membrane perforation, as well as the perceived level of confidence for each diagnosis. The endpoints were diagnostic reliability, diagnostic validity, diagnostic accuracy, and the effects on diagnostic confidence.

### Data analysis/ Statistical methods

The data collected was extracted from the examiners response sheets and tabled in Microsoft Excel for Microsoft 365 (Microsoft Corporation). Continuous measures were described as mean values and standard deviations, and categorical data as absolute and relative frequencies. The 4 groups of the study’s interventions were labeled as follows: the control group of the SME intervention (C1) with ten subjects, the test group of the SME intervention (T1) with ten subjects, the control group of the SMP intervention (C2) with ten subjects and the test group of the SMP intervention (T2) with ten subjects. The rate of true and false diagnoses for each group was calculated as a relative frequency (percentage). To establish the level of diagnostic reliability (inter rater reliability) and diagnostic validity (agreement between examiners' diagnoses and the true diagnosis) of both protocols, chance-adjusted agreement was measured using Gwet AC1 coefficient. This coefficient performs better than Fleiss’ Kappa when high levels of agreement are observed and is considered more robust when the prevalence of one category is very high or very low [56], it avoids the well-established limitations of Kappa that can lead to paradoxical results [57, 58]. The receiver operating characteristic (ROC) analysis was conducted to evaluate the diagnostic accuracy of both the HCL protocol and the saline protocol in predicting the occurrence of membrane lift or perforation.  The combined diagnoses from T1/T2 for the HCL protocol and from C1/C2 for the saline protocol were used as the test variable where the examiners‘ answers were coded as 1 when a lift was diagnosed (positive outcome) and 2 when a perforation was diagnosed (negative outcome) then the true diagnoses were selected as the state variable. To differentiate the effects of the HCL protocol and the saline protocol on the diagnostic validity (detection of SME and SMP) while accounting for the repeated measures of the 10 examiners, a generalized linear mixed model (GLMM) analysis was employed in SPSS. The examiners‘ diagnoses (SME or SMP) were coded 1 when detected and 0 when undetected (binary outcome). This binary outcome was used as the target variable and modelled as a function of the fixed variable (HCL vs. Saline). The analysis accounted for the clustering of observations within examiners using random intercepts to account for the repeated measure design. The related-samples Wilcoxon signed-rank test was used to compare the ratings of the diagnostic confidence level given by the examiners for the test group with those given for the control group while accounting for same subject testing (split mouth design). Finally, The Spearman’s rank correlation coefficient was used to measure the correlation between the type of intervention and the examiners’ level of diagnostic confidence. Statistical analyses were performed using the software SPSS 27.0 (IBM) and AgreeStat360 (Advanced Analytics) and a significance level = 0.01 was used for the hypothesis testing.

## Results

### Clinical documentation of the intervention

**Fig. 5:**
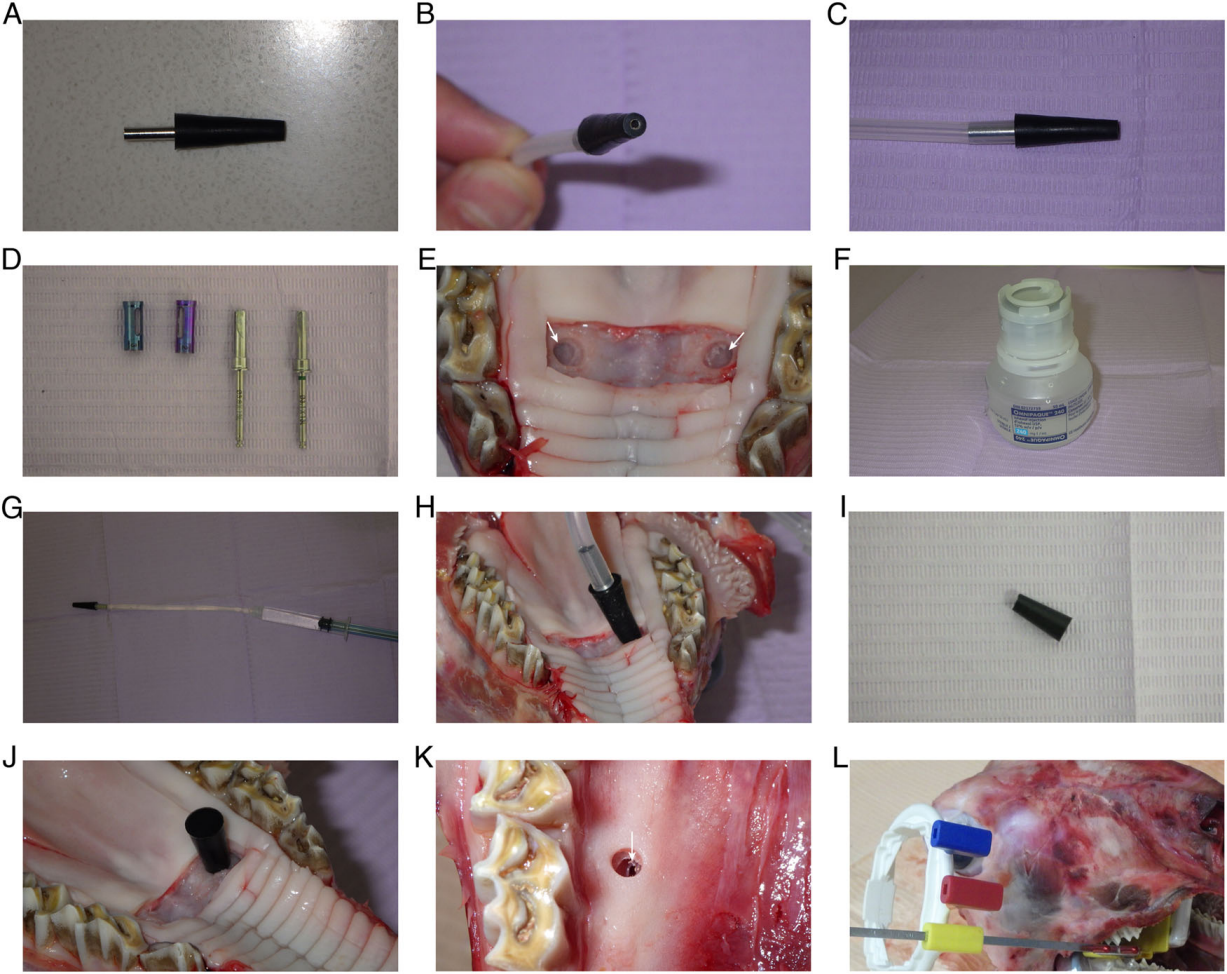
Clinical documentation of the intervention. A Specifically designed silicone retentive end combined with a metallic injection stent. **B** Top view showing how the metallic injection stent supports the silicone retentive end from within to prevent its deformation upon being inserted through the osteotomy. **C** Side view of the retentive end/ injection stent assembly combined with a silicone fluid supply tube. **D** The 3.1 mm and 3.8 mm diameter safe drills from CAS kit (Hiossen Canada) along with the 2 mm and 3 mm drill stoppers used to safely expose the sinus membrane. **E** Following the completion of both right and left side osteotomies, the white arrows show the intact exposed sinus membranes. **F** Omnipaque 240 contrast agent (GE Canada). **G** The specifically designed apparatus combined with a 3 ml graduated syringe loaded with contrast agent. **H** The self-retaining apparatus inserted into one of the osteotomies. **I** Silicone conical plug. **J** Self retaining conical plug inserted into the osteotomy following the fluid injection. **K** White arrow showing artificial perforation in the sinus membrane. **L** Extension cone paralleling device in place prior to periapical radiograph acquisition.

Clinical documentation of the intervention along with the specifically designed apparatus are shown in Fig. 5.

### Radiological documentation of the intervention

**Fig. 6:**
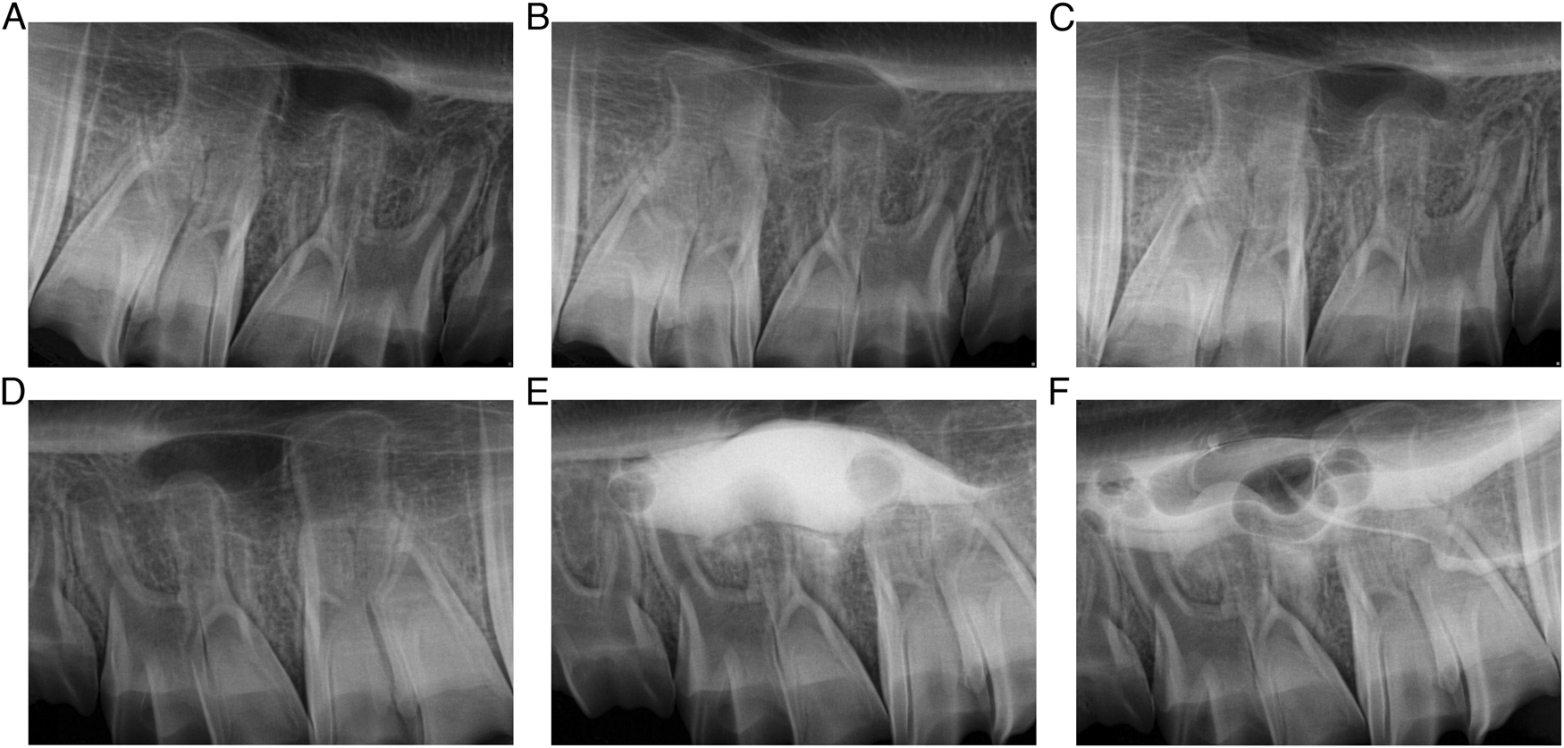
Radiological documentation of the intervention. Series of study periapical radiographs, with the top row representing the control side (right side of sheep maxilla) and the bottom row representing the test side (left side of sheep maxilla) for one of the study subjects. **A**, **D** represent pre-operative radiographs. **B**, **E** represent sinus membrane elevation procedure (intact sinus membrane) on the control side (C1 saline) and the test side (T1 iohexol). **C**, **F** represent sinus membrane perforation procedure (after intentional sinus membrane puncture) on the control side (C2 saline) and the test side (T2 iohexol).

Examples of the preoperative and intraoperative radiographs with and without radiopaque contrast medium are shown in Fig. 6.

### Radiological interpretation/ Evaluation by experienced dentists

**Fig. 7:**
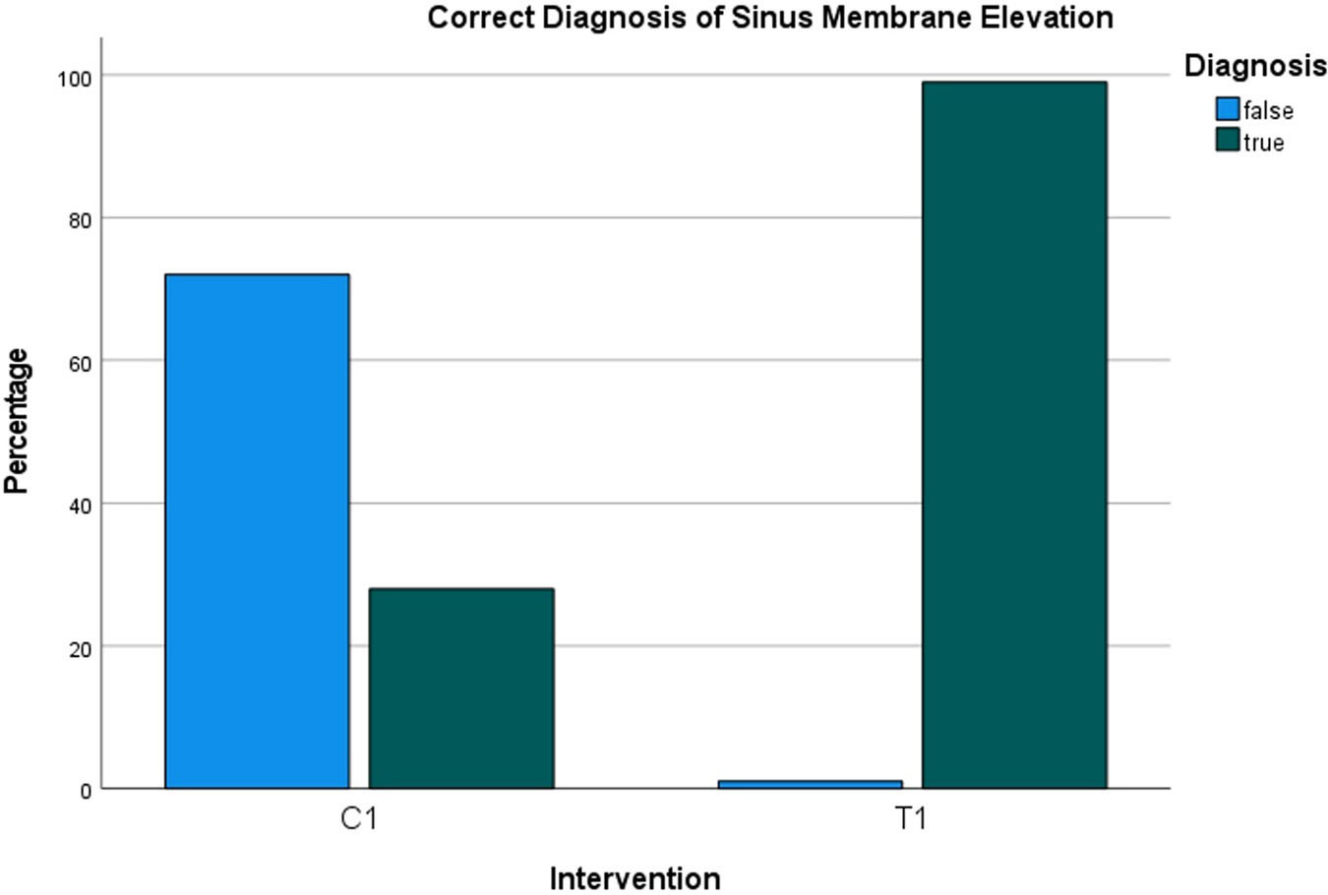
Correct diagnosis rate of sinus membrane elevation. Bar chart showing the detection rate of sinus membrane elevation. In the saline protocol (C1), it was observed in 28% of cases, while in the hydraulic contrast lift protocol (T1), it was observed in 99% of cases.

**Fig. 8:**
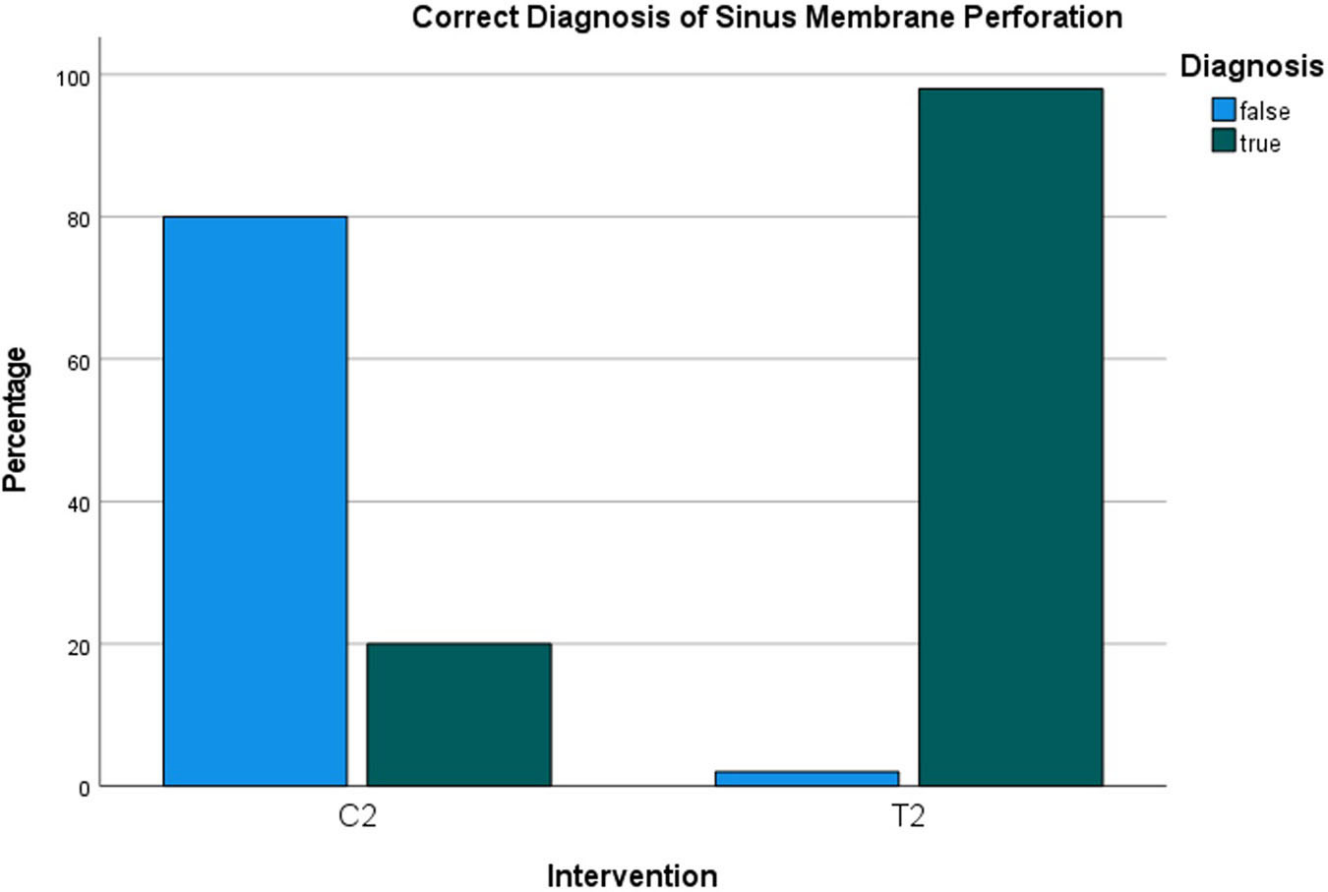
Correct diagnosis rate of sinus membrane perforation. Bar chart showing the detection of sinus membrane perforation was successful in 20% of cases with the saline protocol (C2) and in 98% of cases with the hydraulic contrast lift protocol (T2).

**Fig. 9:**
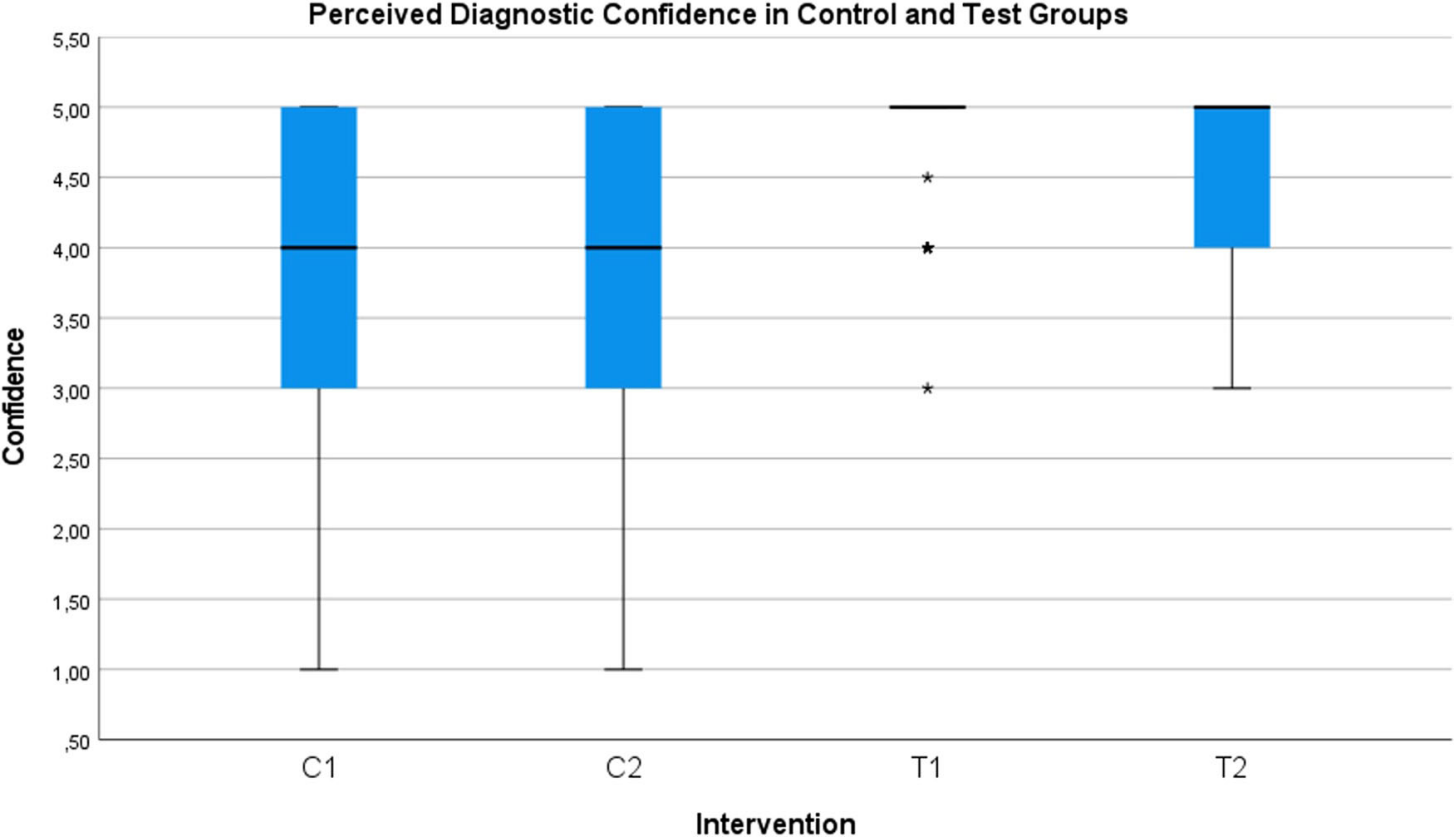
Distribution and central tendency of diagnostic confidence ratings for different interventions. A box and whiskers plot representing the distribution and central tendency of the data obtained from the qualitative analysis of the confidence levels of the examiners. The ratings were obtained when interpreting the radiographs from different interventions, according to the hydraulic contrast lift protocol (T1 and T2) and the saline protocol (C1 and C2), while using a continuous scale between 1 and 5.

In the saline protocol, successful detection of SME was observed in 28% of the interpretations, while in the HCL protocol, it was observed in 99% of the interpretations (Fig. 7). Detection of SMP was successful in 20% of the interpretations with the saline protocol and in 98% with the HCL protocol (Fig. 8). These percentages were obtained from a total of 400 observations from 10 examiners. The average diagnostic confidence ratings for the chosen diagnoses were 3.75 for SME and 3.83 for SMP in the saline protocol, as opposed to 4.90 for SME and 4.62 for SMP in the HCL protocol (Fig. 9, Table 2). The raw data collected from the examiners’ response sheets can be viewed in Appendix 1.

**Table 2:** Confidence level of the ten examiners when interpreting the radiographs of the different study interventions, sinus membrane elevation in the control group (C1), sinus membrane elevation in the test group (T1), sinus membrane perforation in the control group (C2) and sinus membrane perforation in the test group (T2).

	*N*	Mean	SD
**C1**	100	3.75	1.04
**T1**	100	4.90	0.34
**C2**	100	3.83	1.02
**T2**	100	4.62	0.58

*N* Total number of radiological evaluations, *SD* Standard deviation

### Statistical analysis

Regarding the diagnostic reliability, the HCL protocol demonstrated a very high level of agreement among the examiners, with a Gwet AC1 score of 0.98 for SME (*p* < 0.001) and 0.96 for SMP (*p* < 0.001). In contrast, the saline protocol showed poor agreement, with a Gwet AC1 score of 0.04 for SME (*p* = 0.39) and 0.136 for SMP (*p* = 0.04). The agreement was highly statistically significant at the chosen (0.01) level in the HCL protocol for both SME and SMP (Table 3).

**Table 3:** Diagnostic reliability represented by chance adjusted inter rater agreement for the hydraulic contrast lift protocol (T1, T2) and the saline protocol (C1, C2) with Gwet’s AC1 score.

Intervention	Coefficient	Inference		
		SE	99% C.I.	*p*-Value
**C1**	0.04	0.04	−0.11 to 0.19	0.39
**T1**	0.98	0.02	0.91–1.00	< 0.001
**C2**	0.14	0.06	−0.05 to 0.32	0.04
**T2**	0.96	0.03	0.87–1.00	< 0.001

*SE* Standard error, *CI* Confidence interval.

In this study, the diagnostic validity was assessed. Validity analysis with gold standard ratings was performed to establish the agreement between the ratings provided by the examiners and the true diagnoses (Table 4). For the HCL protocol, there was an almost perfect agreement between the ratings and the true diagnosis, with a Gwet AC1 score of 0.98 for SME (*p* < 0.001) and 0.96 for SMP (*p* < 0.001). On the other hand, the saline protocol showed slight disagreement with the true diagnosis, as evidenced by a Gwet AC1 score of −0.05 (*p* = 0.012) for SME and −0.06 for SMP (*p* = 0.001). The agreement was highly statistically significant at the (0.01) level in the HCL protocol for both SME and SMP, while for the saline protocol the disagreement was not significant for SME but was significant for SMP.

**Table 4:** Diagnostic validity represented by chance adjusted agreement between the examiners’ diagnoses and the true diagnoses for the hydraulic contrast lift protocol (T1, T2) and the saline protocol (C1, C2) with Gewt’s AC1 score.

Intervention	Coefficient	Inference		
		SE	99% C.I.	p-Value
**C1**	−0.05	0.01	−0.09 to 0.002	0.012
**T1**	0.98	0.02	0.91–1.00	< 0.001
**C2**	−0.06	0.01	−0.11 to −0.02	0.001
**T2**	0.96	0.03	0.87–1.00	< 0.001

*SE* Standard error, *CI* Confidence interval.

**Fig. 10:**
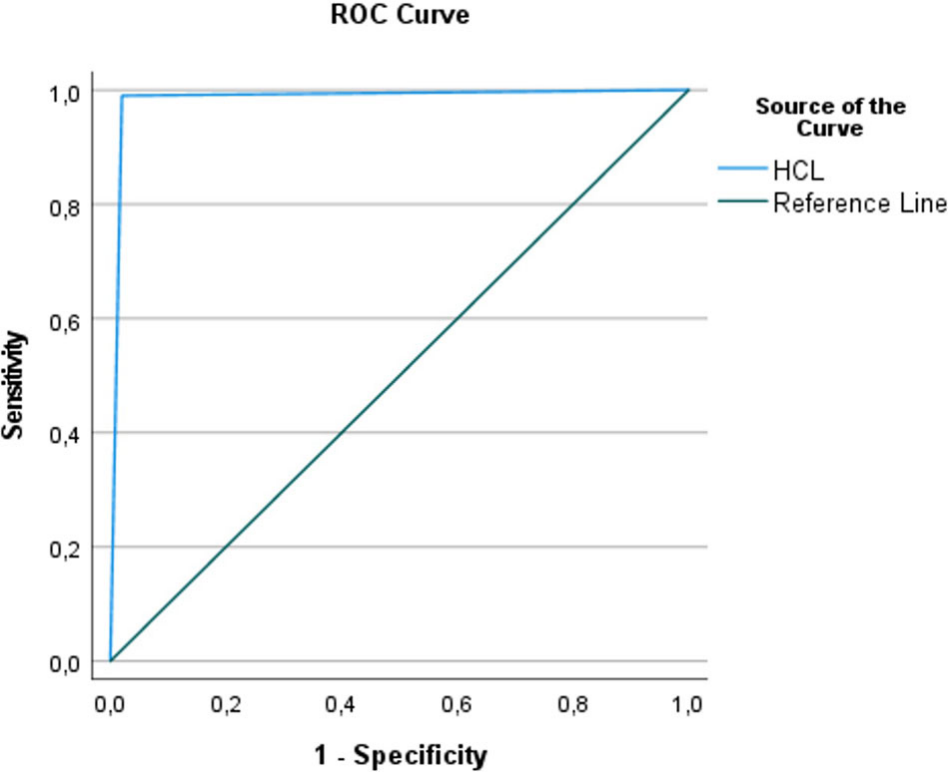
ROC analysis for the hydraulic contrast lift protocol. ROC analysis for the hydraulic contrast lift protocol showing high sensitivity and specificity.

**Fig. 11:**
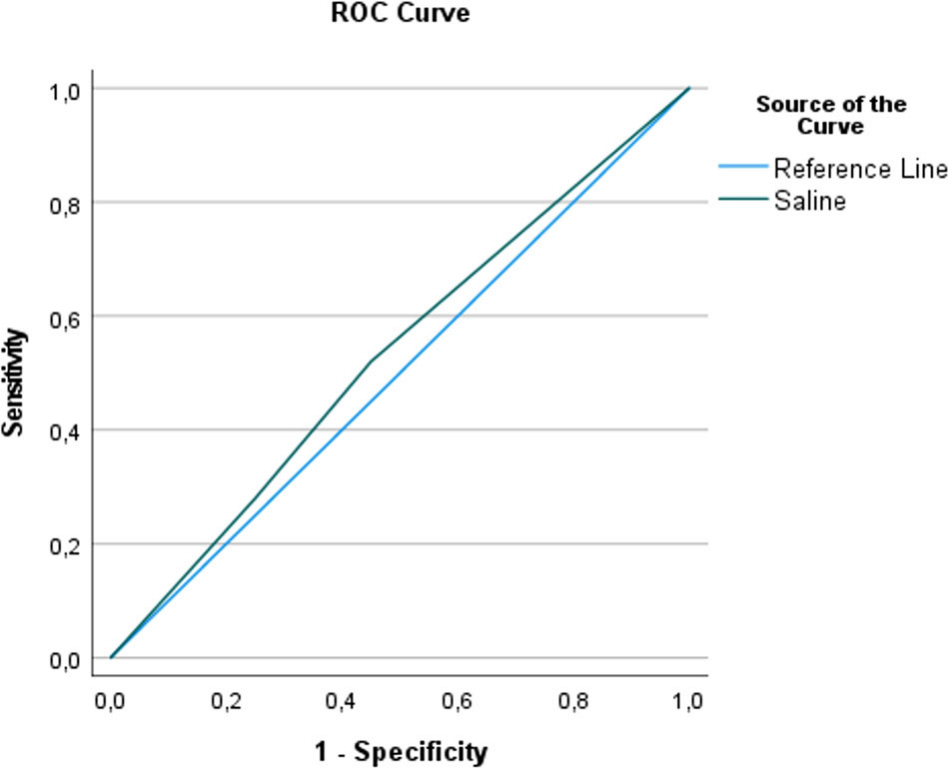
ROC analysis for the saline protocol. ROC analysis for the saline protocol showing low sensitivity and specificity.

Diagnostic accuracy was evaluated. For the HCL protocol, the area under the ROC curve (AUC) was 0.99 (SE = 0.01, *p* < 0.001, 99% CI [0.96, 1.01]) (Fig. 10). The 99% confidence interval suggested that the AUC is significantly different from the null hypothesis value of 0.5. On the other hand, for the saline protocol, the AUC was 0.53 (SE = 0.04, *p* = 0.42, 99% CI [0.43, 0.64]) (Fig. 11). The non-significant *p*-value suggests that the AUC is not significantly different from the null hypothesis value of 0.5.

**Fig. 12:**
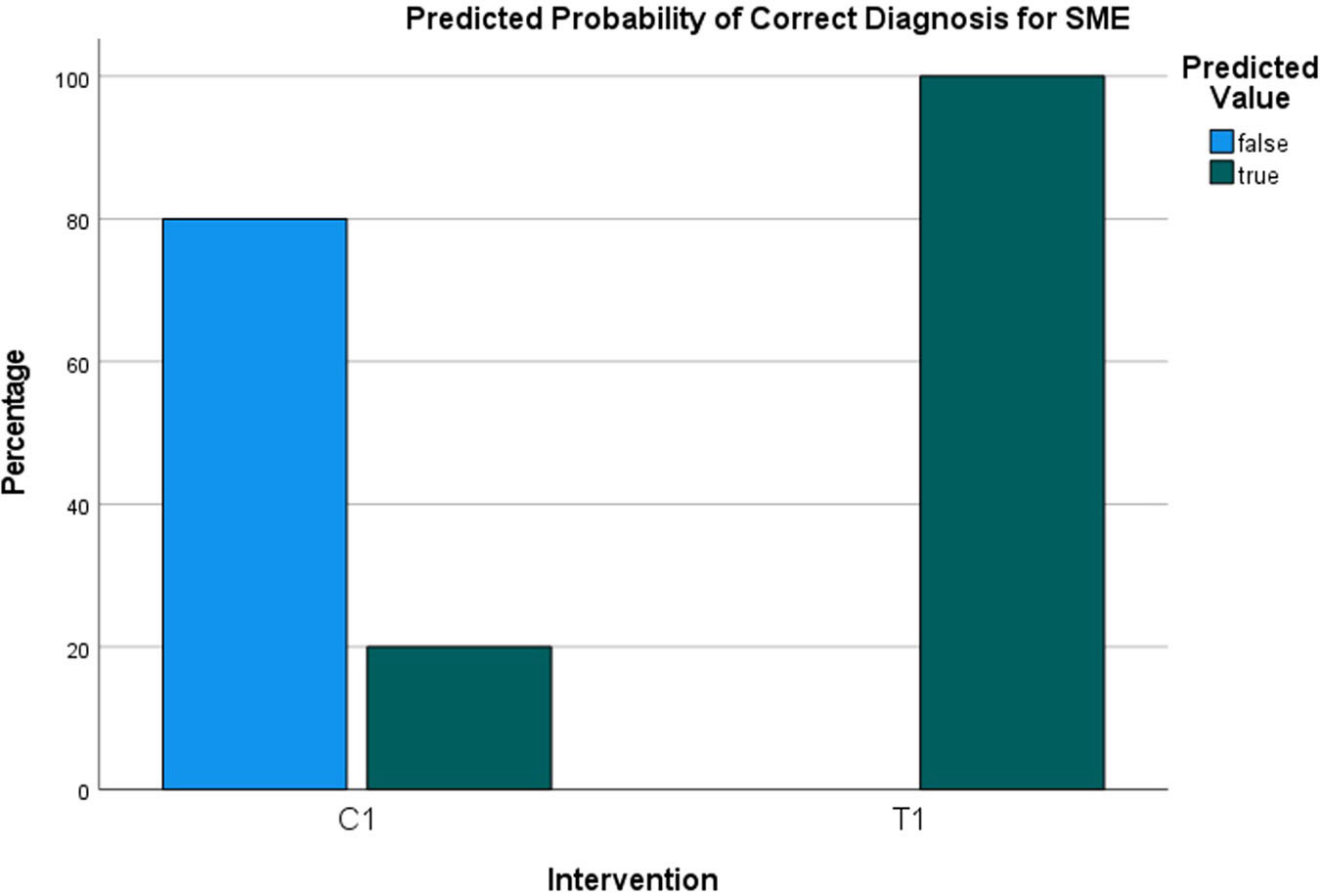
Predicted probability of correct diagnosis for sinus membrane elevation. The generalized linear mixed model analysis to estimate the probability of correct diagnosis for sinus membrane elevation showed an estimated probability of 20% for correct diagnosis in the saline protocol and 100% for correct diagnosis in the HCL protocol according to the logistic regression analysis.

**Fig. 13:**
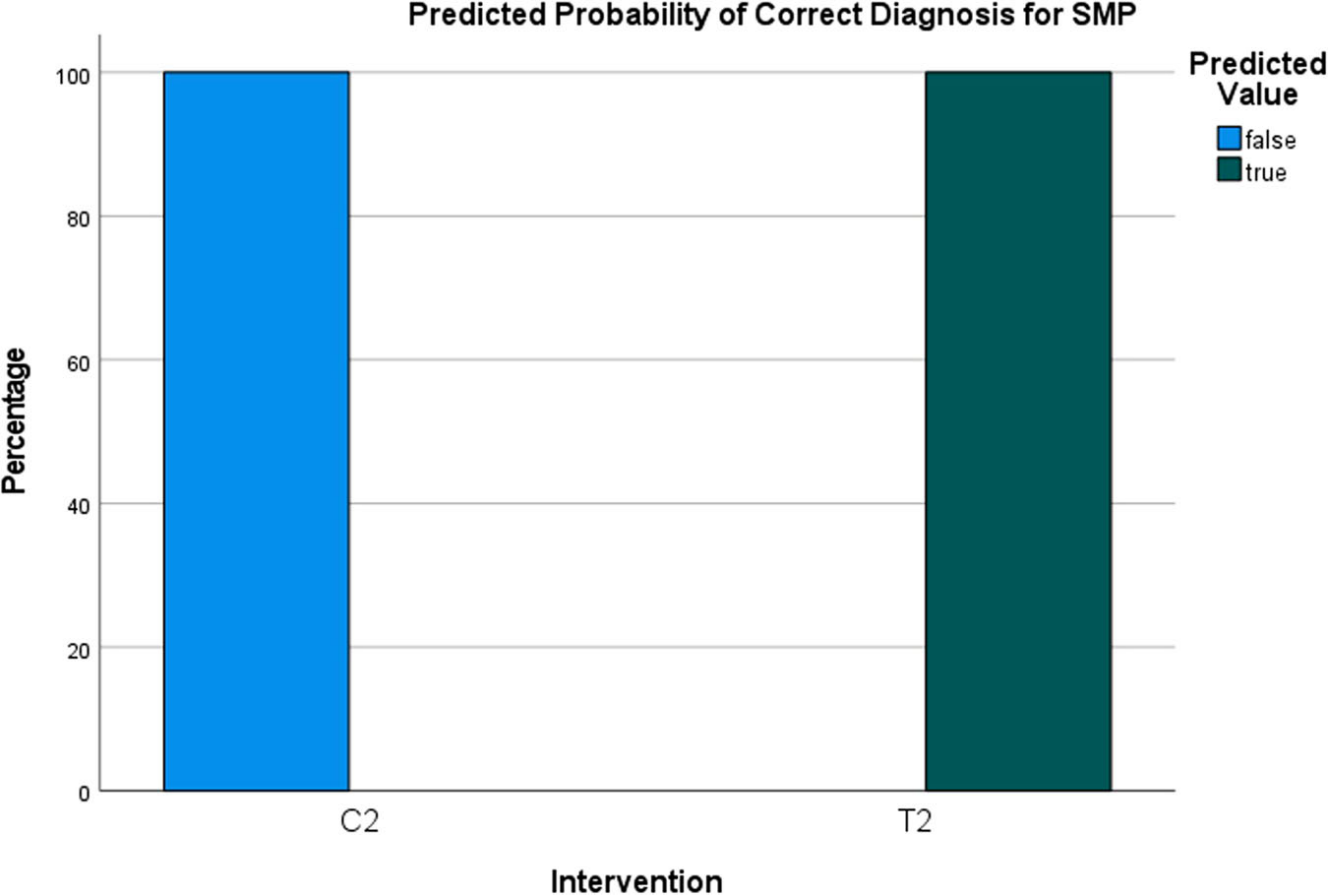
Predicted probability of correct diagnosis for sinus membrane perforation. The generalized linear mixed model analysis to estimate the probability of correct diagnosis for sinus membrane perforation showed an estimated probability of 0% for correct detection (100% false diagnosis) in the saline protocol and 100% for correct detection in the HCL protocol according to the logistic regression analysis.

**Fig. 14:**
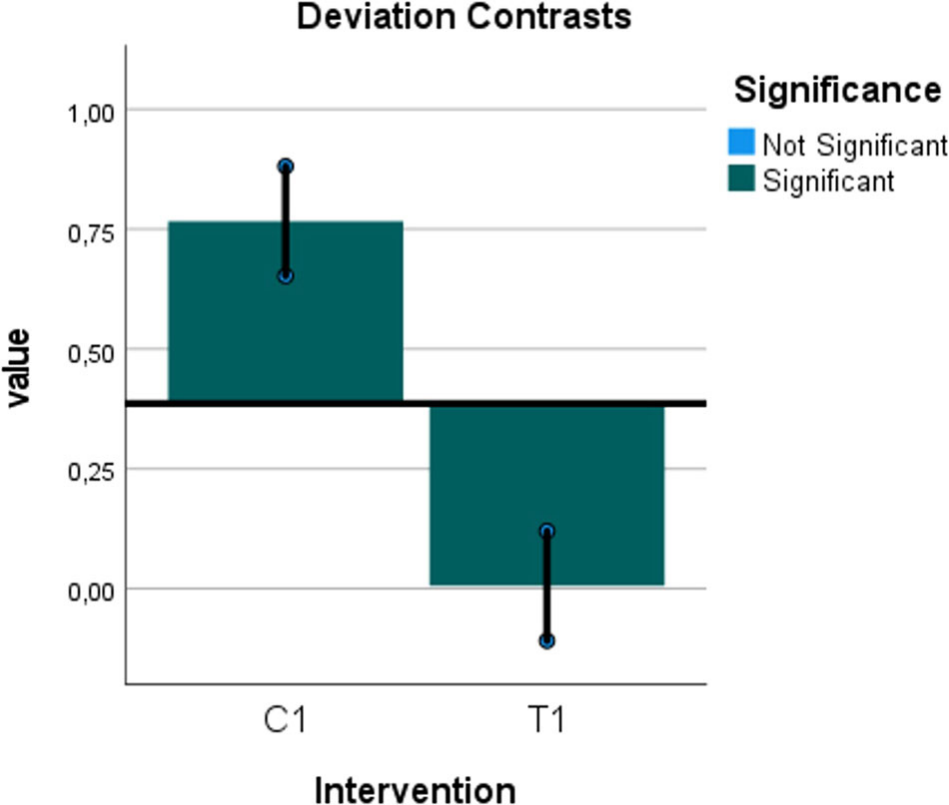
Deviation contrasts graph for the predicted diagnostic validity with sinus membrane elevation. Deviation contrasts graph showing the significant difference in the predicted diagnostic validity between the hydraulic contrast lift protocol and the saline protocol for the correct diagnosis of sinus membrane elevation according to the logistic regression analysis. The horizontal line is the diagnosis overall estimated mean. The vertical bars are the deviation contrasts (diagnosis at each level of intervention minus diagnosis overall). The sequential Bonferroni adjusted significance level is (0.01).

**Fig. 15:**
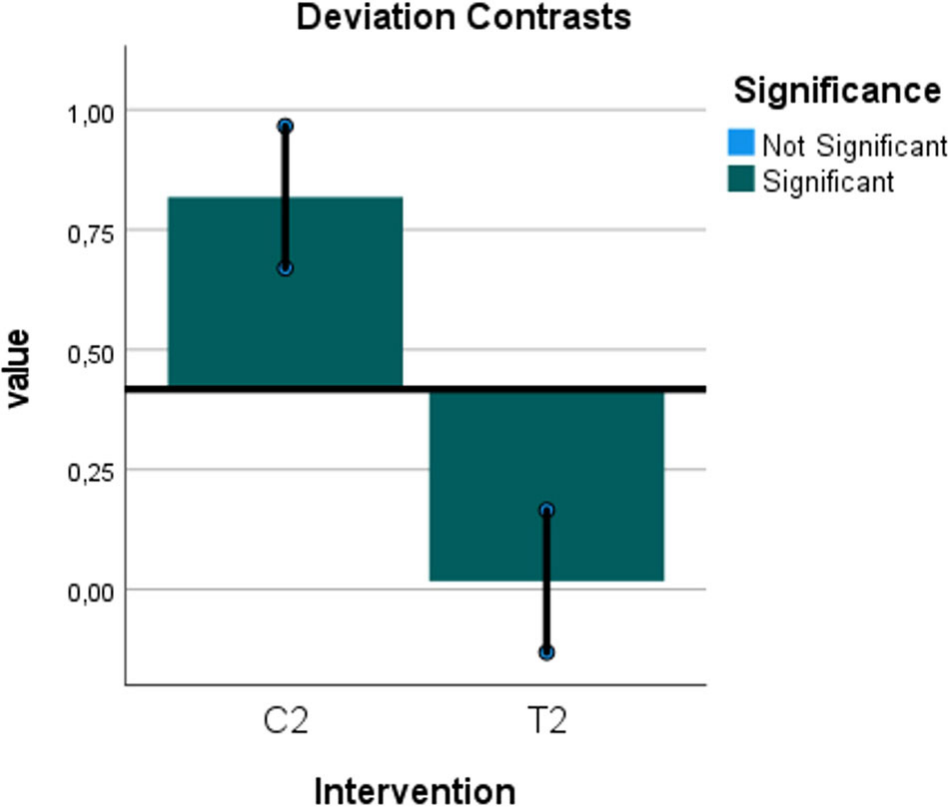
Deviation contrasts graph for the predicted diagnostic validity with sinus membrane perforation. Deviation contrasts graph showing the significant difference in the predicted diagnostic validity between the hydraulic contrast lift protocol and the saline protocol for the correct diagnosis of sinus membrane perforation according to the logistic regression analysis. The horizontal line is the diagnosis overall estimated mean. The vertical bars are the deviation contrasts (diagnosis at each level of intervention minus diagnosis overall). The sequential Bonferroni adjusted significance level is (0.01).

The logistic regression analysis, which considered both protocols (saline vs. HCL), estimated the probability of correct diagnosis of SME to be 20% for the saline protocol and 100% for the HCL protocol (Fig. 12). As for the probability of correct diagnosis of SMP, it was estimated to be 0% for the saline protocol and 100% for the HCL protocol (Fig. 13). The analysis indicated that there was a highly statistically significant difference in the predicted probability of correct diagnosis between the two protocols for SME (OR = 569.38, SE = 1.11, *p* < 0.001) and for SMP (OR = 260.98, SE = 0,78, *p* < 0.001) (Figs. 14 and 15). The odds ratios represent the likelihood of correct diagnosis for the HCL protocol compared to the saline protocol. The large odds ratios indicate a substantial increase in the odds of correct diagnosis with the HCL protocol as compared to the saline protocol.

**Fig. 16:**
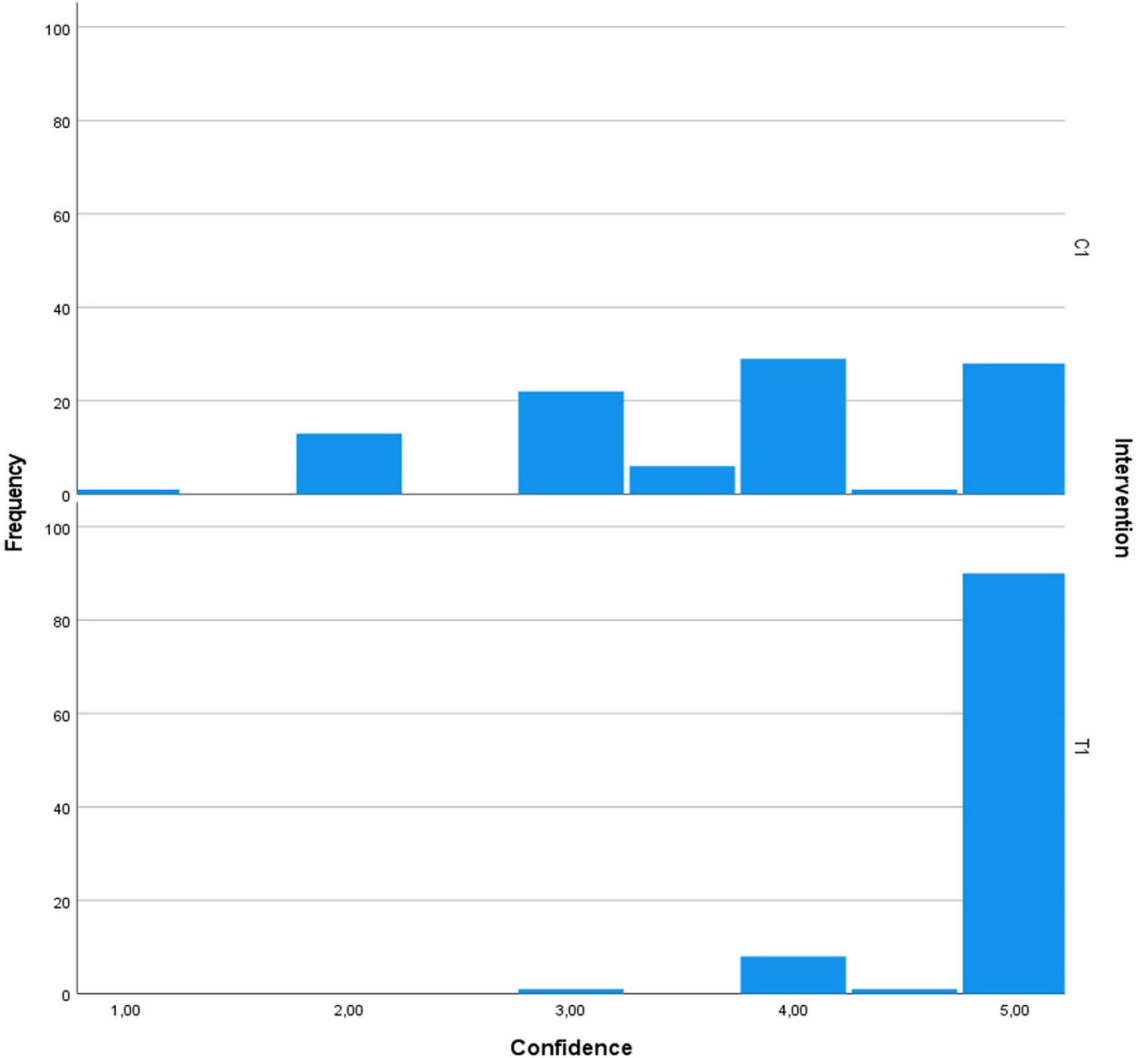
Comparison of the diagnostic confidence distribution for sinus membrane elevation. Bar chart showing the examiners' confidence ratings distribution in sinus membrane elevation intervention in the test group (T1) compared to the control group (C1).

**Fig. 17:**
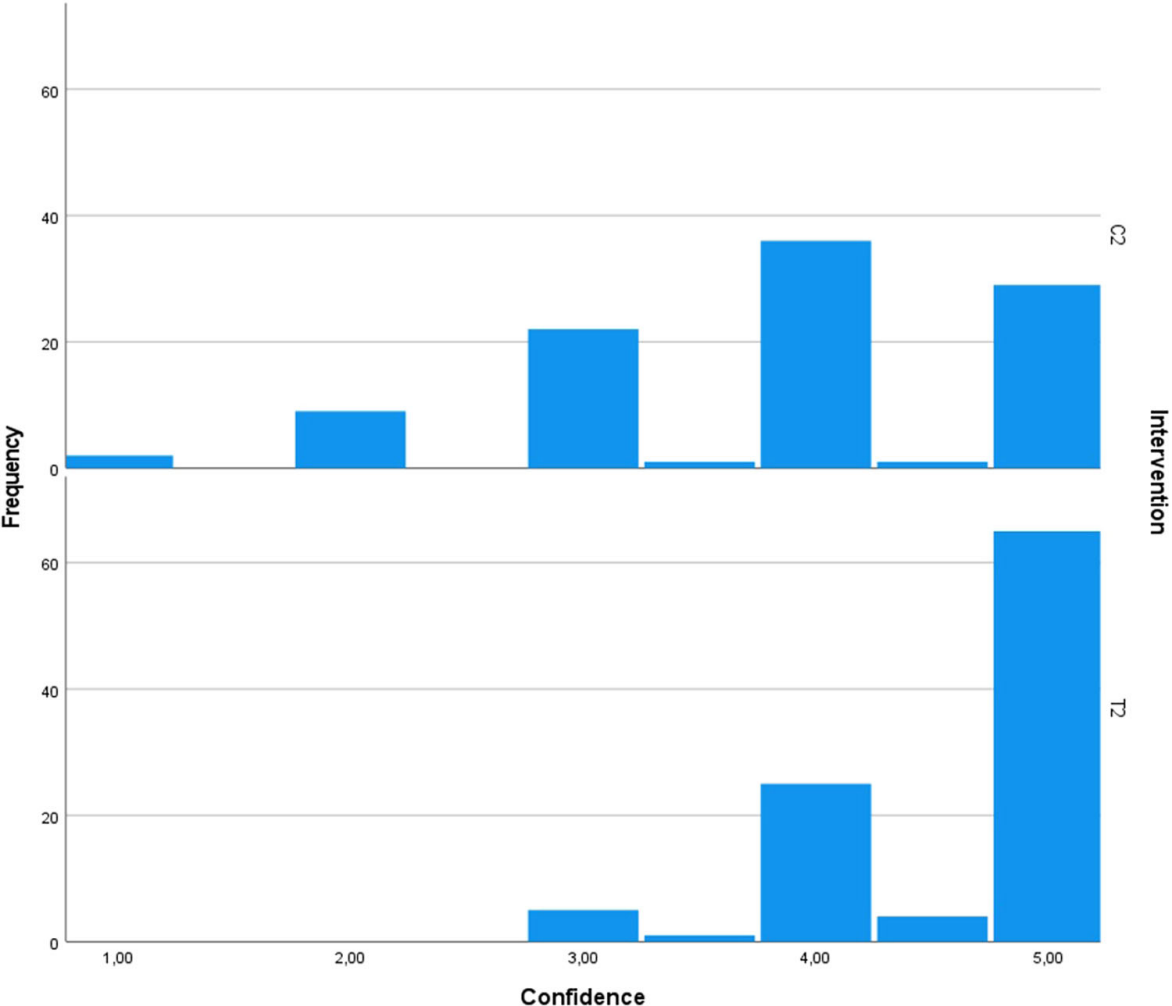
Comparison of the diagnostic confidence distribution for sinus membrane perforation. Bar chart showing the examiners' confidence ratings distribution in sinus membrane perforation intervention in the test group (T2) compared to control group (C2).

**Fig. 18:**
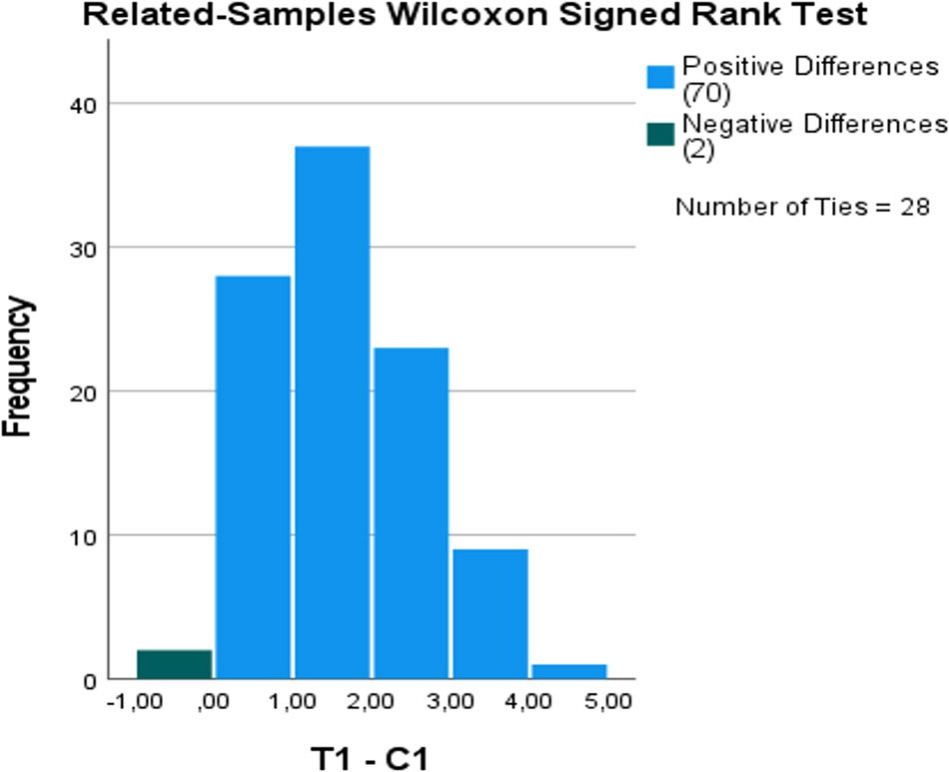
Wilcoxon signed rank test comparing diagnostic confidence ratings for sinus membrane elevation. Wilcoxon signed rank test comparing the examiners' confidence ratings for sinus membrane elevation intervention in the Test group (T1) and the control group (C1) taking into account the split mouth design.

**Fig. 19:**
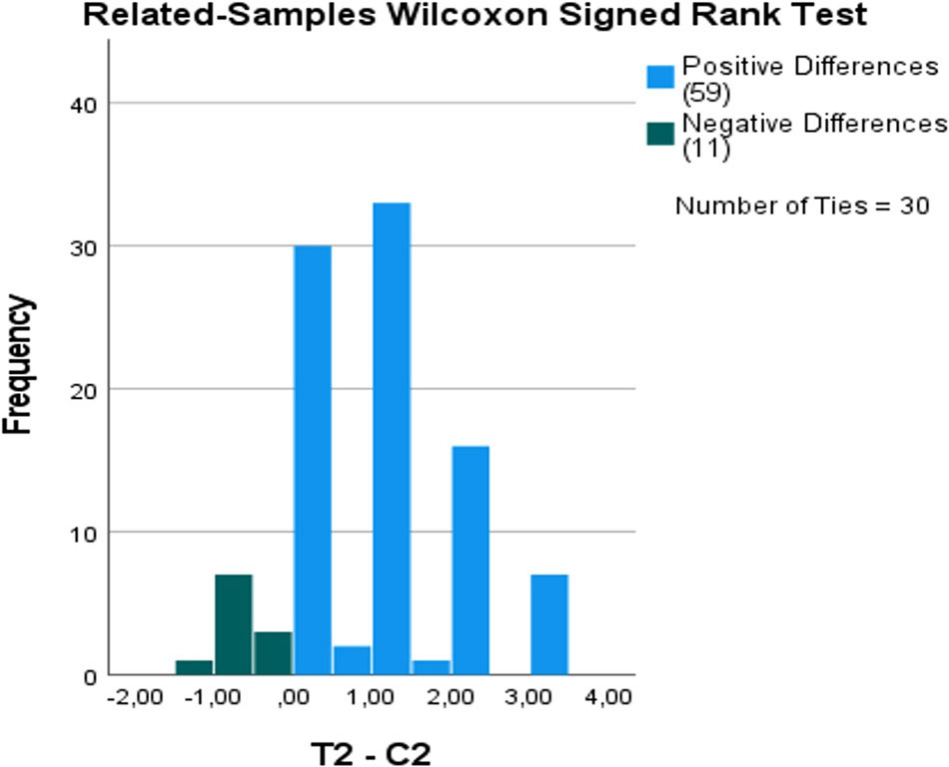
Wilcoxon signed rank test comparing diagnostic confidence ratings for sinus membrane perforation. Wilcoxon signed rank test comparing the examiners' confidence ratings for sinus membrane perforation intervention in the Test group (T2) and the control group (C2) taking into account the split mouth design.

To examine the difference in the examiners’ diagnostic confidence ratings when interpreting the test radiographs as compared to the control radiographs in the split-mouth design, the related-samples Wilcoxon signed rank test was conducted. The test yielded a standardized test statistic of 7.30 for SME and 6.05 for SMP, indicating a substantial difference. Importantly, for both interventions, the asymptotic significance (2-sided test) was (*p* < 0.001), providing strong evidence of a statistically significant difference in confidence ratings (Figs. 16–19).

The relationship between the interventions (HCL protocol or saline protocol) and the examiners’ ratings of diagnostic confidence as a dependent variable was examined. To assess the strength and direction of this relationship, Spearman’s rank correlation coefficient, a nonparametric measure of association, was employed. The analysis revealed a significant positive correlation between the intervention and the confidence ratings for the SME intervention (Spearman’s rho = 0.64, *p* < 0.01) and for the SMP intervention (Spearman’s rho = 0.43, *p* < 0.01). This indicates that as the intervention shifted from the saline protocol to the HCL protocol, there was a concurrent increase in the examiners' diagnostic confidence. The obtained correlation coefficients of 0.64 and 0.43 respectively suggest a moderately strong positive association between the intervention and the confidence ratings.

## Discussion and conclusion

### Discussion

The present study focuses on a rarely described concept in dentistry, the use of contrast media. While Pommer and Watzek [41], and Kim et al. [22], have previously suggested the use of a contrast medium during TSFE using techniques different from the HCL protocol, the value of incorporating this diagnostic aid has never been established. Also, no prior study has explored the ability of any specific diagnostic method to both confirm intact membrane lift and detect membrane perforation intraoperatively during TSFE. In this study, the HCL protocol was experimentally replicated in fresh refrigerated ex-vivo sheep maxillae. It was tested for its merits as a diagnostic test during TSFE, for identifying sinus membrane lift and perforation with the purpose of developing a more reliable and safer technique. It was also tested for its effect on the diagnostic confidence of the operator.

One of the major findings of this study was that the examiners were able to correctly identify successful sinus membrane lifts and membrane perforations more easily and with a much higher rate of correct diagnoses when interpreting the radiographs resulting from the HCL protocol. The examiners reported a significantly higher level of diagnostic confidence that positively correlated with the HCL protocol.

In addition, the diagnostic reliability, diagnostic validity, and diagnostic accuracy of this protocol were shown to be substantial and statistically significant. Also, the differences between both protocols for the detection of both successful lifts and perforations were significant. These findings underscore the superior diagnostic performance and provide strong evidence of the high discriminatory ability of the HCL protocol for radiographic detection of membrane lift and perforation. So, it can be stated that these results establish the scientific basis for the use of this protocol to reduce operator uncertainty when performing TSFE by providing a more reliable detection of membrane perforation.

The reason why this technique provides a higher detection rate is thanks to the ease of visualization of the shape of the sinus membrane on plain radiographs. When the membrane is intact, the contrast medium is enclosed between the flexible membrane and the sinus floor, not being able to escape thanks to the presence of a plug sealing the osteotomy. The membrane is inflated and makes its characteristic convex or dome shape protruding into the lumen of the sinus. On the other hand, that inflated shape would not hold in case of major or even a minor perforation. The more fluid consistency of the contrast medium used, the faster it will escape through a perforation leading to the deflation of that radiopaque balloon and the dispersion of the contrast medium into the sinus lumen leading to a more flattened radiopacity that conforms the most to the shape of the walls and the floor of the sinus.

Another important aspect of that protocol, one that was not tested in this study, is that when the membrane is intact it will still assume the same convex shape on two subsequent radiographs even when the head position changes, whereas if the membrane is perforated the contrast medium would become freely flowing into the sinus lumen and the radiopacity in the sinus will assume different shapes on two subsequent radiographs when the head position is changed.

The reason why diagnostic confidence levels are higher while using this protocol is that dentists are trained to rely heavily on their visual senses in their everyday work while providing treatment or interpreting radiographs. Performing a surgery partly blind is hard to accept for the surgeon and it adds an element of uncertainty. When they can finally visualize an otherwise blind approach, it can easily add a great element of confidence especially if the diagnostic criteria are clear.

There has been no other study about TSFE which was designed with comparable endpoints with the aim to evaluate a diagnostic method or test for its ability to both confirm intact membrane lift and detect membrane perforation. This study is also the first to assess the confidence of the examiners while diagnosing successful membrane lift and perforation. While other studies are trying to avoid perforations, in this study we intentionally made sinus membrane perforations (same number as the elevations) to evaluate if that test can help the assessors match the true diagnosis. This difference in methodology makes it hard to compare our findings with those of other TSFE studies.

For the studies that used tests other than the Valsalva manoeuvre for the detection of perforations, we calculated, when possible, the detection rate from the presented data to compare it to our findings. Nkenke et al. used endoscopy through a punctured hole in the canine fossa in addition to the Valsalva manoeuvre to verify the occurrence of perforations during TSFE along with implant placement in a prospective clinical study. Out of 22 included implants, only one perforation occurred where the Valsalva manoeuvre was negative, demonstrating the limited diagnostic validity thereof, and the only perforation was identified via endoscopy at 100% rate [38]. Elian and Barakat used the endoscope both through a lateral window prepared with flap elevation, and through the crestal osteotomy prepared for immediate implant placement. In this prospective clinical trial, 12 interventions were made, 2 perforations were identified through the crestal osteotomy while only one was identified through the lateral window at rates of 100% and 50% respectively. In both studies, the number of reported perforations was too low to draw a conclusion about the accuracy of identification using this technique. A literature review by Yu et al. to assess the safety and efficacy of endoscope-assisted maxillary sinus elevation concluded that perforations can be detected and managed precisely but high-quality clinical trials are needed to validate the predictability and advantages of this surgical procedure [37]. Though this diagnostic aid allows direct visualization of the sinus membrane, it requires an additional surgical access hole in most cases, needs additional equipment, and requires a learning curve for the operator. Another method was suggested to detect membrane perforations. The Jeder-System (Jeder Sinus-Technology, Vienna, Austria) consists of a pump that generates high hydraulic pressure that pushes back the sinus membrane from the drill upon perforation of the boney floor of the sinus. The pump also monitors the whole procedure by constantly measuring pressure and volume [13]. In a retrospective study by Bruckmoser et al., the Jeder-System was used to perform the lift and detect membrane perforations. The rate of detection of membrane perforation intraoperatively was only 41% (7 out 17 perforations). The remainder of the perforations were not detected intraoperatively by the device nor through the use of the Valsalva manoeuvre, and were detected postoperatively by means of a conventional computed tomography (CT) or CBCT where the graft material was noted in the maxillary sinus lumen [39] demonstrating a lower detection rate for both the device and the Valsalva manoeuvre. Gargalo-Albiol et al. used the operating microscope and the micro-camera during transcrestal sinus floor elevation in an ex vivo study. They concluded that it can detect Schneiderian membrane integrity with greater than 85% accuracy [40], unlike the endoscope method where the viewing device is applied above the sinus membrane which means if the membrane is intact there will be no bleeding and the visibility is good. In this study, the devices were applied on the crestal side of the osteotomy intraorally. This means that the accuracy of detection could be affected by bleeding when applied in real patients.

Pommer and Watzek described the gel pressure technique where Hydroxypropyl Methylcellulose (HPMC) gel and Jopamidol (contrast medium) were mixed in a ratio of 3:1 and used under controlled pressure to elevate the sinus membrane in a human cadaver study, with the aim to evaluate the incidence of perforations and quantify the gain in height in future implant sites. The procedure was monitored through direct vision to verify the occurrence of perforations by removing the orbital floor. No perforations occurred in the study. The ability of that technique to detect membrane perforations was not assessed [41]. In a follow-up prospective clinical trial, Pommer et al. reported one perforation out of 33 cases (3%). It was not described in the study how it was diagnosed but in this case the intervention was aborted [59]. We believe this technique may have some limitations owing to the fact that the contrast medium is mixed with a gel. Firstly, it would have a higher potential for visibility on plain x-ray when undiluted. Secondly, the contrast medium is more fluid without the gel which would allow it to escape through a small perforation and adapt to the shape of the sinus floor or walls thus enabling the operator to detect a perforation more easily whereas the high viscosity and lower flow properties of the HPMC might give the false sense of membrane integrity since it would be more prone to keep its shape leading to a false positive sign of a successful lift.

Kim, Itoh and Kang conducted a preliminary clinical study evaluating the water lift system for sinus floor elevation which has a lateral approach kit and a crestal approach kit. Similar to the discussed studies, the aim of this study was to investigate the capability of the water lift system to reduce the risk of perforating the Schneiderian membrane [22]. For the crestal approach, 66 cases were included. The rate of perforation was 3% (2 out of 66). Interestingly, for this study, saline was not used and a contrast medium (Iobrix 300) was used instead to lift the Schneiderian membrane. Though they did not study the reliability or the validity of this method to diagnose a successful lift, they described an elevated Schneiderian membrane as having a dome shape on a standard X-ray or panoramic imaging and described a perforation as having a collapsed shape of the Schneiderian membrane where the radiographic contrast medium diffused to the inside of the sinus. These descriptions are consistent with our observations.

In this study, the ease of detection by the examiners in both scenarios, lift and perforation, in an animal model with a complex maxillary sinus anatomy compared to that of humans, highlights the great potential of this protocol. Another advantage when using this protocol is that often times, when using the traditional saline based HSFE kits, it is not very easy to tell if the bony floor of the sinus had been perforated even after taking a periapical x-ray. With this approach, when in doubt, the operator could choose to inject some contrast medium into the osteotomy to see if some ballooning of the sinus membrane is detected (bony floor perforated) or if the contrast medium is still enclosed within the confines of the osteotomy (bony floor intact). The criteria provided in the questionnaire for examiners can be used as clear guidelines for interpretation of sinus membrane elevation or perforation.

Using the HCL protocol provides clinicians with a valuable intra-operative tool to confirm membrane lift and detect perforation which allows both the operator and the patient to decide together how to proceed, each operator according to their proficiency and level of comfort along with patient’s input can decide, when perforations occur, whether to abort the procedure or convert it to a traditional lateral window approach that allows direct vision and access to repair the perforation and complete the SFE.

Finally, it is assumed that the volume injected would be between 1 to 5 ml per sinus which constitutes a negligible volume compared to the volumes used routinely in medical imaging. In case a perforation is detected where the contrast medium is intruded beyond the Schneiderian membrane into the sinus lumen, and while the fluidity of the contrast medium would pose no risk of blocking the maxillary sinus ostium compared to an intruded particulate graft material, the ideal outcome is to aim for the complete retrieval of the contrast medium. In the scenario where the clinician and the patient decide to abort the procedure, a small diameter suction tip, when introduced into the osteotomy, should be able to suction out most of the contrast medium provided the maxillary sinus is healthy and the ostium is patent. In the scenario where it is decided to open a lateral window to repair the perforation and complete the SFE the clinician can have access to suction the intruded contrast medium initially through the osteotomy and later directly through the perforated Schneiderian membrane via the lateral window. In case the complete retrieval is not achieved, and since the contrast medium used for this study is indicated for oral use and has a good palatability [44, 51], it is deemed safe if the remainder of the fluid is not retrieved since it will eventually be cleared out via the patent sinus ostium in the nasopharynx and either spit out by the patient or ingested.

### Limitations of this study

A limitation of this study was the fact that air bubbles would sometimes become trapped within the contrast medium, along with the use of radiolucent plugs. Both had the effect of causing areas of radiolucency within the area of radio opacity in both contexts, as can be seen on (Fig. 5e, f), and it was important to explain to the examiners during the calibration session that the mere presence of such radio-lucencies is not enough to be interpreted as a perforation and that they should instead look at the outer shape of the radio-opaque entity where the dome/ bell shape implied the ballooning of the sinus membrane (fluid cannot escape, lifted) and the flatness and irregular shape implied the deflation of the sinus membrane (fluid escaped, perforated). Another limitation is that in this ex-vivo model, there is no bleeding associated with the dissection of the sinus membrane, it remains to be seen if such bleeding could hamper the radio-opacity of the contrast medium when injected below the sinus membrane. A third limitation that the use of this technique would not be possible in subjects with allergy to iodine, but this could be easily overcome by substituting the iohexol with a non iodinated contrast medium with similar viscosity and flow properties.

## Conclusion

Following the HCL protocol, the use of a radiographic contrast medium can be an invaluable diagnostic aid to reliably confirm lift and detect perforation during TSFE intraoperatively prior to bone graft application in addition to improving the diagnostic confidence of the operator while relying on Periapical radiographs. This technique has great potential for clinical application, more precisely in reducing complications following TSFE.

### Future outlook

This technique provides a more reliable alternative for the Valsalva manoeuvre and the mirror fog up test as an intraoperative test and can become the new standard procedure for TSFE/ HSFE due to low cost and improved patient safety. Future clinical trials are recommended to study the effects of contrast medium use on the healing process and on both bone graft and implant integration.

### Supplementary information

Appendices 1-2

## Data Availability

The data that support the findings of this study are openly available in figshare at 10.6084/m9.figshare.24153330.v1 and 10.6084/m9.figshare.24187755.v1.
