# Supplementary material for: Diagnostic reliability and accuracy of the hydraulic contrast lift protocol in the radiographic detection of sinus lift and perforation: ex vivo randomized split-mouth study in an ovine model
Source: BDJ Open. 2024 Jan 31;10:6. doi: 10.1038/s41405-024-00188-6 (PMC10830460; doi:10.1038/s41405-024-00188-6)
Supplement: Supplementary file 1 — Appendices 1-2 [file 41405_2024_188_MOESM1_ESM.pdf]

Appendix 1 Raw data collected from the examiners' response sheets and tabled in Microsoft excel. The “I” designates the interpretation (diagnosis), and the “C” designates the confidence level when choosing the interpretation for each of the examiners

Subject	Intervention	Ex 1 I	Ex 1 C	Ex 2 I	Ex 2 C	Ex 3 I	Ex 3 C	Ex 4 I	Ex 4 C	Ex 5 I	Ex 5 C	Ex 6 I	Ex 6 C	Ex 7 I	Ex 7 C	Ex 8 I	Ex 8 C	Ex 9 I	Ex 9 C	Ex 10 I	Ex 10 C
SHEEP2	C2	3	4	3	5	3	5	3	5	1	5	2	3	2	2	3	5	3	5	2	3
SHEEP6	C1	1	5	1	4	1	3	3	4	2	3	1	3	3	2	2	3	2	2	3	4
SHEEP15	C2	3	4	3	5	3	5	3	4	1	4	2	3	2	3	3	5	3	4	2	4
SHEEP7	C1	3	4	3	4	3	5	3	5	2	3	2	4	1	2	3	5	3	2	1	4
SHEEP4	C1	1	5	1	4	2	3	3	4	2	3	1	2	3	2	2	3	2	4	2	3.5
SHEEP3	T1	1	5	1	5	1	5	1	5	1	5	1	5	1	4	1	5	1	4	1	5
SHEEP2	C1	3	4	3	4	3	5	3	5	1	5	2	3	2	2	3	5	3	5	1	3.5
SHEEP5	T2	2	5	2	5	2	4	2	5	1	5	2	5	2	3	2	5	2	5	2	5
SHEEP5	C1	1	5	3	5	2	3	3	4	1	3	1	3	2	2	2	3	3	5	3	3
SHEEP9	T2	2	5	2	4	2	3.5	2	5	2	5	2	5	2	4	2	5	2	5	2	5
SHEEP7	T1	1	5	1	5	1	5	1	5	1	5	1	3	1	5	1	5	1	5	1	5
SHEEP3	C2	1	4	3	5	3	4	3	4	3	5	1	4	1	1	3	3	3	5	3	3
SHEEP6	C2	3	4	3	3	2	2	3	4	1	5	1	3	3	1	3	3	2	2	1	4
SHEEP15	C1	3	4	3	5	3	5	3	5	2	5	1	3	2	2	3	5	3	2	2	3.5
SHEEP4	C2	1	5	3	3	1	4	3	4	3	4	1	4	2	2	2	3	2	2	2	4
SHEEP14	T2	2	5	2	5	2	4.5	2	5	2	4	2	4	2	4	2	5	2	4	2	5
SHEEP6	T1	1	5	1	5	1	5	1	5	1	5	1	5	1	5	1	5	1	5	1	5
SHEEP7	C2	3	4	2	3	3	4	3	5	2	3	1	3	2	2	3	5	3	5	1	4
SHEEP3	C1	1	5	3	5	1	3.5	3	4	3	4	1	4	2	2	2	3	3	3	2	3.5
SHEEP10	T1	1	5	1	5	1	4.5	1	5	1	5	1	5	1	5	1	5	2	5	1	5
SHEEP9	C1	3	4	1	4	3	4.5	3	4	2	4	1	3	1	2	2	3	3	4	3	4
SHEEP15	T1	1	5	1	5	1	5	1	5	1	5	1	5	1	4	1	5	1	5	1	5
SHEEP4	T1	1	5	1	5	1	5	1	5	1	5	1	5	1	4	1	5	1	5	1	5
SHEEP14	C2	1	4	3	4	3	5	3	5	1	4	1	3	3	2	3	5	3	4	1	3.5
SHEEP15	T2	2	3	2	4	2	4	2	5	2	4	2	3	2	4	2	5	2	5	2	5
SHEEP10	C2	1	4	3	5	3	5	3	5	1	4	1	3	2	2	3	3	3	4	1	4
SHEEP9	T1	1	5	1	5	1	5	1	5	1	5	1	5	1	5	1	5	1	5	1	5
SHEEP10	T2	2	4	2	5	2	4.5	2	5	2	5	2	3	2	5	2	5	2	5	2	4.5
SHEEP9	C2	3	4	3	5	3	5	3	5	2	4	2	3	2	3	3	5	3	4	3	3
SHEEP2	T1	1	5	1	5	1	4	1	5	1	5	1	4	1	5	1	5	1	5	1	5
SHEEP5	T1	1	5	1	5	1	5	1	5	1	5	1	5	1	4	1	5	1	5	1	5
SHEEP2	T2	2	5	2	4	2	5	2	5	2	5	2	4	2	5	2	5	2	5	2	5
SHEEP14	C1	1	4	3	5	3	5	3	5	1	5	2	3	1	1	3	5	3	4	3	4
SHEEP4	T2	1	5	2	4	2	4.5	2	5	2	4	2	4	2	4	2	5	2	5	2	5
SHEEP5	C2	1	4	3	5	3	4.5	3	4	1	4	1	3	1	2	3	3	3	5	2	4
SHEEP6	T2	2	5	2	5	2	5	2	5	2	4	2	3	2	4	2	5	2	5	2	5
SHEEP14	T1	1	5	1	5	1	5	1	5	1	5	1	4	1	5	1	5	1	5	1	5
SHEEP3	T2	2	5	2	4	2	5	2	5	2	5	2	5	2	4	2	5	2	5	2	5
SHEEP10	C1	1	4	3	5	3	4.5	3	5	1	4	1	3	1	2	3	3	3	5	3	4
SHEEP7	T2	2	4	2	5	2	5	2	5	2	4	2	5	2	4	2	5	2	5	2	5

Appendix 2 Questionnaire for Examiners. For each periapical radiograph of the posterior region of the Ovine Maxilla provided to you please choose the *ONE* option that best describes what you see by marking the box on the left side of the table with an "X" then indicate the level of confidence for each of the chosen interpretations with any numerical value between 1 and 5, decimal values can be included, with 1 being the lowest and 5 being the highest possible value

**Choice**	**Radiographic interpretation that best describes what I see on x-ray number *(x)***	**Confidence**
	**Successful lift** of the sinus membrane as evidenced by an **enclosed entity/radio opacity** or a **well-defined entity/radio opacity** that **exhibits a dome** or **semilunar** shape or **ballooning/tenting** of the sinus membrane.	
	**Perforation** of the sinus membrane as evidenced by an **ill-defined entity/radio opacity** or a **diffuse entity/radio opacity** that **does not exhibit a uniform** shape or has an **irregular** shape or **that loosely follows** the shape of the sinus floor and/or walls.	
	**Absence of such entities** or **No appreciable difference from normal radiographic anatomy** of the Ovine Maxilla.	
